# Medication non-adherence and self-inflicted violence behaviors among 185,800 patients with schizophrenia in the community: a 12-year cohort study

**DOI:** 10.1186/s12916-024-03354-7

**Published:** 2024-03-25

**Authors:** Chuanlong Zuo, Xianmei Yang, Xiangrui Wu, Ruoxin Fan, Jun Liu, Hu Xiang, Yang Li, Xing Zhao, Xiang Liu, Yuanyuan Liu

**Affiliations:** 1 Department of Epidemiology and Biostatistics, West China School of Public Health and West China Fourth Hospital, Sichuan University, No.17, Section 3, Renmin South Road, Chengdu, Sichuan 610041 China; 2 Sichuan Mental Health Center, The Third Hospital of Mianyang, No. 190, Jiannan East Road, Mianyang, Sichuan 621000 China; 3 Department of Health Behavior and Social Medicine, West China School of Public Health and West China Fourth Hospital, Sichuan University, No.17, Section 3, Renmin South Road, Chengdu, Sichuan 610041 China

**Keywords:** Medication adherence, Schizophrenia, Suicide, Self-harm, Community

## Abstract

**Background:**

Despite the importance of medication adherence in treatment effectiveness, little is known about the association between medication non-adherence and self-inflicted violence behaviors. We aimed to assess whether medication non-adherence increased the risk of self-inflicted violence behaviors among schizophrenics in communities (hypothesis 1) and whether the dose–response relationship existed (hypothesis 2).

**Methods:**

This 12-year cohort study in western China recruited 292,667 community-dwelling schizophrenics. The proportion of regular medication (PRM) was calculated by dividing the time of “regular adherence” by the total time of antipsychotic treatment during follow-up period as an indicator of medication adherence. For hypothesis 1, medication adherence was designated as a binary variable with a threshold of 0.8 (PRM); for hypothesis 2, medication adherence was specified as five-category and continuous variables, respectively. Inverse probability weighting and mixed effects Cox proportional hazards models were conducted for confounders control and survival analyses.

**Results:**

One hundred eighty-five thousand eight hundred participants were eligible for the final analyses, with a mean age of 47.49 years (SD 14.55 years), of whom 53.6% were female. For hypothesis 1, the medication non-adherence group (PRM < 0.8) had a lower risk of suicide (HR, 0.527, 95% CI, 0.447–0.620), an increased risk of NSSI (HR, 1.229, 95% CI, 1.088–1.388), and non-significant risk of attempted suicide compared with adherence group (PRM ≥ 0.8). For hypothesis 2, the lowest medication adherence (PRM < 0.2) was associated with increased risks of suicide attempt (HR, 1.614, 95% CI, 1.412–1.845), NSSI (HR, 1.873, 95% CI, 1.649–2.126), and a decreased risk of suicide (HR, 0.593, 95% CI, 0.490–0.719). The other non-adherence groups had lower risks for all three self-inflicted violence behaviors. The associations between medication adherence in continuous-variable and three outcomes were consistent with the categorical medication adherence results.

**Conclusions:**

Almost no medication taken as prescribed was associated with an increased risk of suicide attempt and NSSI. However, medication adherence did not appear to prevent completed suicide. Besides, patients with moderate adherence had a lower incidence of suicide attempt and NSSI. These findings highlight the need for a more detailed portrayal of medication adherence and the need to be vigilant for suicide intent in schizophrenics with good medication adherence who may be overlooked previously.

**Supplementary Information:**

The online version contains supplementary material available at 10.1186/s12916-024-03354-7.

## Background

Schizophrenia is a serious, chronic psychiatric disorder, characterized by long-term, recurrent psychotic symptoms, and functional impairment [1]. The global burden of the study 2019 estimated that 12.2% of disability-adjusted life-year for mental disorders could be attributed to schizophrenia [2]. A meta-analysis suggests that schizophrenia contributes to 14.5 years of potential life lost [3]. Suicide is one of the leading causes of premature death in schizophrenics [4]. An American study including 668,836 participants found that the risk of suicide was 4.5 times higher in people with schizophrenia compared to the general population [5]. The lifetime prevalence of suicide attempt and non-suicidal self-injury (NSSI) for schizophrenics is 26.8% and 49%, respectively, much higher than the general population [6–9]. Furthermore, suicide attempt and NSSI are strong predictors of subsequent suicide [10]. Consequently, prevention of self-inflicted violence behaviors (i.e., suicide, suicide attempt, and NSSI) is of great importance to reduce premature death and disease burden in individuals with schizophrenia.

Antipsychotic medications have long been the primary treatment for schizophrenia [11]. Literature suggests that psychotropic drugs are effective in reducing the risk of self-inflicted violence behaviors [12–14]. Although medication adherence is important in treatment effectiveness and as a modifiable factor, little is known about the association between medication non-adherence and self-inflicted violence behaviors. Some studies have investigated this relationship [15–22]. However, most of the epidemiological evidence focuses on the exploration of factors influencing self-inflicted violence behaviors [16–21]. Limited studies have identified medication non-adherence as a primary exposure [15, 22]. The results of these studies were ambiguous and based on short follow-up and limited sample size. Large-scale prospective epidemiological evidence is still scarce. In addition, the study populations were predominantly from high-income countries. Evidence was challenging to generalize to populations from low- and middle-income countries, where culture, lifestyles, and mental health resources are disparate substantially from those in high-income countries. Besides, medication adherence in previous studies was usually defined as dichotomous or trichotomous variables. Given the complexity and changeability of medication adherence, it should be portrayed and categorized in more detail [23]. Furthermore, these studies have also focused on adverse clinical outcomes (i.e., hospitalization, relapse, and remission) and suicide (whether successful or not), whereas self-inflicted violence behaviors have not been systematically inspected. Methodologically, previous studies using traditional statistical approaches instead of counterfactual frameworks limited the identification and control of confounders.

To address the above research gaps, we conducted an investigation based on a large-scale 12-year cohort study from western China. The purpose of this study was to examine two hypotheses: (1) compared to medication adherence, whether medication non-adherence increases the risk of self-inflicted violence behaviors (hypothesis 1), and (2) whether the dose–response relationship that the lower levels of medication adherence gradually elevate the risk of self-inflicted violence behaviors exists (hypothesis 2). To our knowledge, this is the first study focusing on the association between medication non-adherence and self-inflicted violence behaviors among patients with schizophrenia in the community, and the findings may fill the gap in the related field and provide a reference for future community self-harm and suicide prevention.

## Methods

### Study design and participants

We did a population-based, prospective cohort study using routinely recorded data from the integrated management information platform for severe mental disorders in western China. The platform was established in 2006 to facilitate integrated hospital-community patient management. The platform captured basic sociodemographic at baseline and dynamic health information about patients at least every 3 months. More details of the study design and participants have been described previously [24].

We were granted access to de-identified data covering the period from May 1, 2006 (the earliest available), to December 31, 2018. This study strictly followed the standards for utilizing anonymized data and was approved by the local ethics committee. We constructed the cohort as comprising all individuals with schizophrenia with the 3-character ICD-10 code of F20. A total of 292,667 schizophrenia patients aged 10 to 100 were enrolled. Inclusion criteria were patients enrolled in community management and receiving antipsychotic drug medication. Individuals with logical errors (i.e., follow-up earlier than 2006, age greater than 100 years or less than 10 years, duration of illness greater than age), medication adherence recordings of more than one, and missing information on key variables (age, sex, ethnic, marital status, urbanicity, education level, economic situation, family history of psychiatric illness, and date of diagnosis) were excluded. The screening flow chart is shown in Additional file Fig. S1. This study followed the Strengthening the Reporting of Observational Studies in Epidemiology (STROBE) reporting guideline.

### Outcomes

The primary outcomes were completed suicide (i.e., death caused by self-directed injurious behavior with an intent to die as a result of the behavior), suicide attempt (i.e., self-injury behavior with intent to die), and NSSI (i.e., self-injury behavior without intent to die, distinguish the behavior from suicide attempts) during the follow-up period. Individuals were followed from the date of first follow-up until the earliest of: documentation of the outcome, the date of last follow-up, or death other than suicide.

Suicide was identified by the patient’s family. If a patient is found dead during a follow-up visit, the patient’s family is asked about the information related to the death. The cause of death was recorded in the platform, including physical disease, suicide, homicide, accidental death, complications, and other causes of death. Suicide attempt and NSSI were identified by professionals who examined the patients as well as interviewed the patients and their family members.

### Exposure

Medication non-adherence was identified as the exposure of interest. It was documented in the platform by mental health professionals, based on the patient- or their family- reported compliance with the antipsychotic medication regimen from the preceding follow-up to the current one. In practice, medication adherence is divided into 4 categories: (1) regular adherence, denoting the action of taking medication as prescribed; (2) intermittent adherence, indicating the situations of not taking medication as prescribed, either in frequency or quantity; (3) non-adherence, referring to the situation where the patients have not taken the medication at all; (4) no medication, is designated for the scenario in which medication is not necessary according to the prescription. Each time a patient was followed up, the patient’s medication adherence was recorded for that follow-up period, so most patients had more than 1 adherence record. Therefore, based on the above classifications, we calculated the proportion of regular medication (PRM; range, 0–1.00) as an indicator of medication adherence in the subsequent analysis. Specifically, the PRM was calculated by determining the proportion of “regular adherence” days to the total number of days with antipsychotic treatment from the initial follow-up to the event of interest or censoring. For hypothesis 1, the PRM was classified as a binary variable including medication adherence (PRM ≥ 0.8, reference group) and medication non-adherence (PRM < 0.8), with the threshold of 0.8 being chosen based on previous studies [25, 26]. In further analysis for hypothesis 2, we classified the PRM into 5 levels: P1 (PRM < 0.2), P2 (0.2 ≤ PRM < 0.4), P3 (0.4 ≤ PRM < 0.6), P4 (0.6 ≤ PRM < 0.8), and P5 (PRM ≥ 0.8, reference group). Furthermore, PRM was directly used as a continuous variable to explore possible nonlinear dose–response relationship. Because more than one outcome occurred in some patients and different outcomes occurred at different times in the same patient, PRMs were not calculated identically for different outcomes, at which point we formed separate cohorts for suicide, suicide attempt, and NSSI for the specific analyses that followed.

### Potential confounders

We selected potential confounders a prior guided by directed acyclic graphs (DAGs) for medication non-adherence and self-inflicted violence outcomes (Additional file Figure S2). Sociodemographic characteristics included age, sex, ethnic, marital status, urbanicity, education level, and economic situation. Clinical characteristics involved family history of psychiatric illness and duration of illness. Self-inflicted violence behaviors history characteristics were history of suicide attempt or NSSI at baseline. History of suicide attempt or NSSI during follow-up was additionally considered in the suicide cohort. Definition and encoding are in Additional file Table S1.

### Statistical analysis

Data were analyzed from January 1, 2023, to October 5, 2023.

We summarized the characteristics of participants using means and SDs or frequencies with proportions. To compare the differences in covariates of exposure groups, standardized mean difference (SMD), *t*-tests, and *χ*^2^ tests were conducted.

To control for potential confounders, inverse probability weighting was performed. We estimated the propensity score using a binomial logistic regression model (for hypothesis 1), a multinomial logistic regression model, or a linear model (for hypothesis 2). Since unstable weights could lead to more extreme weights that inflate the variance of the estimator, stabilized weights were computed as the marginal probability of the observed groups with different levels of medication adherence divided by the propensity scores for the corresponding groups [27]. SMD was used to check whether the distributions of covariates in the weighted cohorts were balanced or not. An SMD of 0.1 or less was considered to be ideally balanced.

The Kaplan–Meier method was used to estimate the cumulative incidence curves for weighted populations. Weighted Cox proportional hazards models were used to investigate the risk of self-inflicted violence behaviors in people with different levels of medication adherence. Accounting for the hierarchical structure of data arising from regional clustering, analyses of weighted population included region as a random effect. We also assessed the non-linear dose-relationship of PRM with the incidence of self-inflicted violence behaviors with cubic restricted spline function (Additional file Supplementary Method). The proportional hazards assumption was assessed using Schoenfeld residuals. For the models that violated this assumption, hazard ratios (HRs) will be interpreted as weighted averages of the time-varying HRs throughout the follow-up [28]. To address the non-proportional hazards, we also reported restricted mean survival time, which was defined as the average event-free time up to a prescribed time in a given population, calculated as the area under the survival curve [29]. The truncation point was the minimum of each group’s last event time.

The robustness of the primary results was tested in sensitivity analyses. First, we reanalyzed patients with more than 1 year of follow-up to mitigate reverse causation. Second, as the PRM calculation using medication adherence with too few follow-up visits may be unstable, the misclassification bias was reduced by excluding patients with fewer than 5 follow-up visits. Third, considering that no medication was also a form of compliance behavior, we re-defined the PRM by calculating the proportion of “regular adherence” and “no medication” days to the total number of days from the initial follow-up to the event of interest or censoring. Fourth, to quantitively assess the level of unmeasured confounding, we computed *E*-values for each of the models. The *E*-values indicate what the HR would need to be for an unmeasured confounder, or set of confounders, to explain away the associations observed in the models [30].

Finally, to assess whether associations varied across subgroups, we fitted weighted Cox hazards proportional models with mixed effects stratified by sex, age, and urbanity.

All *P* values were 2-tailed and less than 0.05 was considered statistically significant. All statistical analyses were done with R (version 4.1.1).

## Results

**Fig. 1:**
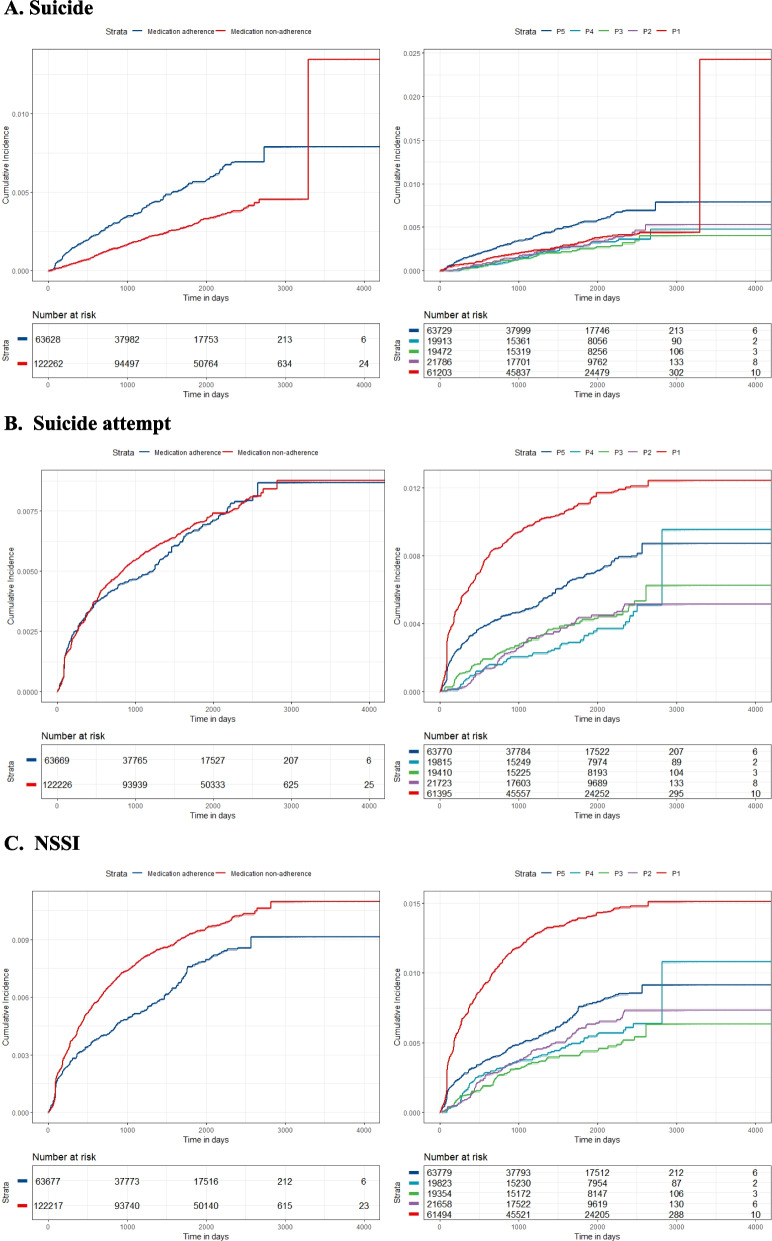
Cumulative incidence of self-inflicted violence behaviors with different levels of medication adherence. The cumulative incidence curves were weighted by the stabilized weights. The curves in the left column are for hypothesis 1 (binary medication adherence) and the curves in the right column are for hypothesis 2 (medication adherence for the five classifications)

One hundred eighty-five thousand eight hundred participants with schizophrenia were eligible for the final analyses. Before weighting, the study population had a mean age of 47.49 years (SD 14.55 years) and comprised 53.6% females. The cohort had a median follow-up of 4.08 years and a maximum follow-up of 12.35 years. During follow-up, 573 (0.3%), 1112 (0.6%), and 1392 (0.7%) participants have engaged in suicide, suicide attempt, and NSSI, respectively. The prevalence of self-inflicted violence behaviors showed significant between-group and regional differences (Additional file Table S2-S3). The weighted risk curves are presented in Fig. 1. More details of the suicide cohort are presented in Table 1. Weighting resulted in an improved balance of selected covariates between the exposed and control groups assessed by using SMD (Additional file Table S4-S8). The baseline characteristics of the suicide attempt and NSSI cohorts were similar to the suicide cohort before or after weighting (Table 1 and Additional file Table S4-S8).

**Table 1:** Characteristics for the suicide cohort of participants

	**Total (*n* = 185,800)**	**Unweighted**				**IPW**^a^			
		**Medication adherence (*n* = 63,860)**	**Medication non-adherence (*n* = 121,940)**	***P* value**	**SMD**	**Medication adherence (*n* = 63,627.51)**	**Medication non-adherence (*n* = 122,261.95)**	***P* value**	**SMD**
**Sociodemographic characteristics**									
Age, mean (SD), years	47.49 (14.55)	44.70 (14.33)	48.95 (14.46)	< 0.001	0.296	47.33 (14.47)	47.37 (14.63)	0.647	0.002
Sex				< 0.001	0.026			0.667	0.002
Male	86,158 (46.4)	30,163 (47.2)	55,995 (45.9)			29,410.2 (46.2)	56,651.3 (46.3)		
Female	99,642 (53.6)	33,697 (52.8)	65,945 (54.1)			34,217.3 (53.8)	65,610.6 (53.7)		
Ethnic				0.849	0.001			0.839	0.001
Han	184,174 (99.1)	63,297 (99.1)	120,877 (99.1)			63,047.2 (99.1)	121,159.5 (99.1)		
Minorities	1626 (0.9)	563 (0.9)	1063 (0.9)			580.3 (0.9)	1102.5 (0.9)		
Marital status				< 0.001	0.169			< 0.001	0.054
Never married	51,157 (27.5)	19,052 (29.8)	32,105 (26.3)			16,638.0 (26.1)	33,706.7 (27.6)		
Married	115,556 (62.2)	37,925 (59.4)	77,631 (63.7)			40,841.5 (64.2)	75,643.4 (61.9)		
Widowed	8857 (4.8)	2225 (3.5)	6632 (5.4)			2657.3 (4.2)	6053.7 (5.0)		
Divorced	10,230 (5.5)	4658 (7.3)	5572 (4.6)			3490.7 (5.5)	6858.1 (5.6)		
Urbanicity				< 0.001	0.596			0.502	0.003
Rural	150,631 (81.1)	41,745 (65.4)	108,886 (89.3)			51,516.0 (81.0)	98,825.1 (80.8)		
Urban	35,169 (18.9)	22,115 (34.6)	13,054 (10.7)			12,111.5 (19.0)	23,436.9 (19.2)		
Education level				< 0.001	0.511			< 0.001	0.028
Primary school or lower	123,532 (66.5)	32,709 (51.2)	90,823 (74.5)			42,227.8 (66.4)	81,309.6 (66.5)		
Middle and high school	58,526 (31.5)	28,444 (44.5)	30,082 (24.7)			20,169.5 (31.7)	38,112.7 (31.2)		
College/university or higher	3742 (2.0)	2707 (4.2)	1035 (0.8)			1230.3 (1.9)	2839.6 (2.3)		
Economic situation				< 0.001	0.301			0.954	< 0.001
Poverty	120,102 (64.6)	35,237 (55.2)	84,865 (69.6)			41,050.8 (64.5)	78,862.6 (64.5)		
Non-poverty	65,698 (35.4)	28,623 (44.8)	37,075 (30.4)			22,576.7 (35.5)	43,399.3 (35.5)		
** Clinical characteristics**									
Family history of psychiatric illness				< 0.001	0.061			0.945	< 0.001
Yes	8737 (4.7)	3551 (5.6)	5186 (4.3)			2984.7 (4.7)	5726.0 (4.7)		
No	177,063 (95.3)	60,309 (94.4)	116,754 (95.7)			60,642.8 (95.3)	116,536.0 (95.3)		
Duration of illness									
mean (SD), years	12.78 (11.17)	11.61 (10.42)	13.39 (11.49)	< 0.001	0.163	12.39 (11.08)	12.66 (11.14)	< 0.001	0.024
< 10	92,619 (49.8)	34,415 (53.9)	58,204 (47.7)	< 0.001	0.157	32,508.0 (51.1)	61,783.9 (50.5)	0.068	0.015
10 ~ 19	48,991 (26.4)	16,595 (26.0)	32,396 (26.6)			16,500.7 (25.9)	32,013.7 (26.2)		
20 ~ 29	27,489 (14.8)	8542 (13.4)	18,947 (15.5)			9201.5 (14.5)	17,652.2 (14.4)		
≥ 30	16,701 (9.0)	4308 (6.7)	12,393 (10.2)			5417.3 (8.5)	10,812.1 (8.8)		
**History of self-inflicted violence behaviors**									
History of suicide attempt at baseline				< 0.001	0.034			0.032	0.014
Yes	851 (0.5)	259 (0.4)	592 (0.5)			252.3 (0.4)	596.5 (0.5)		
No	172,068 (92.6)	58,829 (92.1)	113,239 (92.9)			58,915.7 (92.6)	113,188.7 (92.6)		
Unknown	12,881 (6.9)	4772 (7.5)	8109 (6.6)			4459.5 (7.0)	8476.7 (6.9)		
History of NSSI at baseline				< 0.001	0.040			0.070	0.013
Yes	809 (0.4)	198 (0.3)	611 (0.5)			240.0 (0.4)	560.2 (0.5)		
No	171,471 (92.3)	58,714 (91.9)	112,757 (92.5)			58,711.1 (92.3)	112,801.7 (92.3)		
Unknown	13,520 (7.3)	4948 (7.7)	8572 (7.0)			4676.4 (7.3)	8900.0 (7.3)		
History of suicide attempt during follow-up				< 0.001	0.022			0.736	0.002
Yes	1112 (0.6)	313 (0.5)	799 (0.7)			389.7 (0.6)	730.0 (0.6)		
No	184,688 (99.4)	63,547 (99.5)	121,141 (99.3)			63,237.8 (99.4)	121,531.9 (99.4)		
History of NSSI during follow-up				< 0.001	0.052			0.745	0.002
Yes	1392 (0.7)	300 (0.5)	1,092 (0.9)			465.0 (0.7)	914.5 (0.7)		
No	184,408 (99.3)	63,560 (99.5)	120,848 (99.1)			63,162.5 (99.3)	121,347.4 (99.3)		

^a^Inverse probability weighting

Table 2 demonstrates the associations of medication non-adherence with self-inflicted violence behaviors. Against hypothesis 1, medication non-adherence was associated with a reduced risk of suicide (HR, 0.527, 95% CI, 0.447–0.620), an increased risk of NSSI (HR, 1.229, 95% CI, 1.088–1.388), and non-significant risk of suicide attempt (HR, 1.003, 95% CI, 0.882–1.140).

**Table 2:** Association between 2 levels of medication adherence and self-inflicted violence behaviors during follow-up period (for hypothesis 1)

Self-inflicted violence behaviors	Statistics	Medication adherence	Medication non-adherence	*P* value
**Suicide**	Hazard ratio (95% CI)^a^	Ref	0.527 (0.447, 0.620)	< 0.001
	Restricted mean survival time at 2738 days (95% CI)			
	Difference—days	Ref	5.452 (3.767, 7.137)	< 0.001
	Ratio	Ref	1.002 (1.001, 1.003)	< 0.001
**Suicide attempt**	Hazard ratio (95% CI)^a^	Ref	1.003 (0.882, 1.140)	0.960
	Restricted mean survival time at 2570 days (95% CI)			
	Difference—days	Ref	− 0.708 (− 2.615, 1.206)	0.470
	Ratio	Ref	1.000 (0.999, 1.001)	0.470
**NSSI**	Hazard ratio (95% CI)^a^	Ref	1.229 (1.088, 1.388)	< 0.001
	Restricted mean survival time at 2570 days (95% CI)			
	Difference—days	Ref	− 4.643 (− 6.700, − 2.587)	< 0.001
	Ratio	Ref	0.998 (0.997, 0.999)	< 0.001

^a^The model included region as a random effect. The variance (standard deviation) of the random effect of region was 0.04 (0.20) for suicide, 0.12 (0.35) for suicide attempt, and 0.31 (0.55) for NSSI

Table 3 provides the results of associations of 5 levels of medication adherence with self-inflicted violence behaviors (for hypothesis 2). It shows that 4 levels of medication non-adherence (i.e., P1–P4) were all associated with a significantly lower risk of completed suicide. For the other two outcomes, the lowest medication adherence (P1) was associated with an increased risk of suicide attempt (HR, 1.614, 95% CI, 1.412–1.845) and NSSI (HR, 1.873, 95% CI, 1.649–2.126). However, groups P2, P3, and P4 had significantly reduced risks for both suicide attempt and NSSI. In other words, we did not find any evidence of a linear dose–response consistent with hypothesis 2. The directions of restricted mean survival time were consistent with the hazard ratios of all self-inflicted behaviors.

**Table 3:** Association between 5 levels of medication adherence and self-inflicted violence behaviors during follow-up period (for hypothesis 2)

Self-inflicted violence behaviors	Statistics	P5	P4	P3	P2	P1
**Suicide**	Hazard ratio (95% CI)^a^	Ref	0.489 (0.361, 0.664)^***^	0.443 (0.322, 0.609)^***^	0.563 (0.429, 0.740)^***^	0.593 (0.490, 0.719)^***^
	Restricted mean survival time at 2534 days (95% CI)					
	Difference—days	Ref	5.345 (3.415, 7.275)^***^	5.908 (4.021, 7.796)^***^	4.822 (2.868, 6.776)^***^	4.066 (2.351, 5.780)^***^
	Ratio	Ref	1.002 (1.001, 1.003)^***^	1.002 (1.002, 1.003)^***^	1.002 (1.001, 1.003)^***^	1.002 (1.001, 1.002)^***^
**Suicide attempt**	Hazard ratio (95% CI)^a^	Ref	0.495 (0.377, 0.649)^***^	0.589 (0.458, 0.757)^***^	0.570 (0.448, 0.727)^***^	1.614 (1.412, 1.845)^***^
	Restricted mean survival time at 2345 days (95% CI)					
	Difference—days	Ref	6.806 (4.853, 8.758)^***^	5.081 (2.926, 7.236)^***^	5.479 (3.441, 7.517)^***^	− 9.169 (− 11.561, − 6.777)^***^
	Ratio	Ref	1.003 (1.002, 1.004)^***^	1.002 (1.001, 1.003)^***^	1.002 (1.001, 1.003)^***^	0.996 (0.995, 0.997)^***^
**NSSI**	Hazard ratio (95% CI)^a^	Ref	0.711 (0.566, 0.893)^**^	0.576 (0.451, 0.737)^***^	0.745 (0.604, 0.919)^**^	1.873 (1.649, 2.126)^***^
	Restricted mean survival time at 2345 days (95% CI)					
	Difference—days	Ref	3.742 (1.429, 6.055)^**^	5.119 (2.898, 7.339)^***^	3.051 (0.732, 5.370)^*^	− 13.834 (− 16.424, − 11.245)^***^
	Ratio	Ref	1.002 (1.001, 1.003)^**^	1.002 (1.001, 1.003)^***^	1.001 (1.000, 1.002)^*^	0.994 (0.993, 0.995)^***^

^*^P value < 0.05, ^**^P value < 0.01, ^***^P value < 0.001

^a^The model included region as a random effect. The variance (standard deviation) of the random effect of region was 0.04 (0.19) for suicide, 0.13 (0.36) for suicide attempt, and 0.31 (0.56) for NSSI

**Fig. 2:**
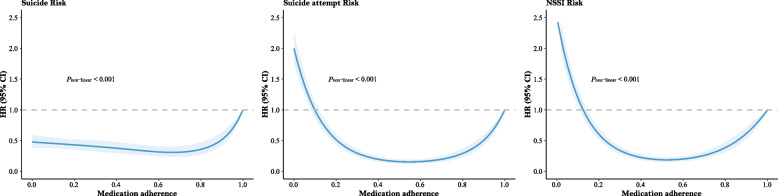
Non-linear relationship between PRM and self-inflicted violence behaviors. HR, hazard ratio; CI, confidence interval; NSSI, non-suicidal self-injury; PRM, the proportion of regular medication. PRM of 1 was served as the reference group. Inverse probability weighting was according to age, sex, ethnic, marital status, urbanicity, education level, economic situation, family history of psychiatric illness, duration of illness, and history of suicide attempt or NSSI at baseline. History of suicide attempt or NSSI during follow-up was additionally considered in the suicide cohort. *P*_non-linear_ < 0.05 indicated that significant non-linear relationship

The dose–response curves showed a non-linear relationship between PRM and self-violence outcomes (Fig. 2). The findings were consistent with those of the five categories of medication adherence, i.e., as medication adherence increased, the risk of self-violence behaviors first decreased and then increased. The risk of suicide attempt and NSSI was higher at the worst level of medication adherence (i.e., PRM close to 0) than at the best level of medication adherence (i.e., PRM of 1), and the opposite was observed for suicide.

Sensitivity analyses were provided in Additional file Table S9-S14. The associations of medication non-adherence with self-inflicted violence were steady. *E*-values were explained away by unmeasured confounders by HRs ranging from 1.76 to 3.94. (Additional file Table S15).

In the subgroup analyses, the association between medication non-adherence and self-inflicted violence behaviors was similar between male–female, rural–urban, and across age groups (Additional file Table S16-S26). There were no significant time trends in the Schoenfeld residuals for most models (Additional file Table S27).

## Discussion

In this 12-year prospective cohort study, we systematically investigated the associations between medication non-adherence and self-inflicted violence behaviors among over 180,000 people with schizophrenia in the community. The research design and rigorous analyses aimed to obviate confounding and selection bias that was not contemplated in previous studies. The results showed better medication adherence might not be sufficient to decrease the risk of suicide. However, for suicide attempt and NSSI, the lowest medication adherence was associated with an elevated incidence, whereas the remaining non-adherence groups had reduced risks.

### Compared with other studies

Firstly, our observation that the medication non-adherence group had a lower risk of suicide than the medication adherence group was different from what has been reported in previous studies from high-income countries. For hypothesis 1, previous case–control studies found that completed suicides had a higher percentage of medication non-adherence than controls [16, 18–20]. As time sequence was not considered in these studies, we conducted a prospective cohort study and performed sensitivity analysis on participants with more than 1 year of follow-up to ensure the temporal order of exposure and outcome. As for hypothesis 2, findings from the Canadian health care database showed good medication adherence (medication possession ratio ≥ 0.8, MPR) was associated with a lower risk of successful or attempted suicide in Quebec compared with poor medication adherence (MPR < 0.5) but no significant association in Saskatchewan [22]. In addition, the incidence of successful or attempted suicide had no statistical discrepancy between good and moderate (0.5 ≤ MPR < 0.8) medication adherence. However, this study did not distinguish between suicide attempt and completed suicide and had little information on individual-level covariates. Additionally, the MPR in the Canadian study only reflects access to medication. Differently, we regarded completed and attempted suicide as two distinct outcomes and control a priori for more person-level confounders based on DAGs. Besides, the PRM in our study reflects the extent to which patients maintain their medication regimen, and the categorization thresholds of the two studies were not the same. These may explain the difference between our results and previous studies.

Secondly, our findings about suicide attempt were inconsistent with previous studies. Results from 10 European countries found that medication non-adherence was associated with suicide attempt among outpatients [15]. In our analyses for hypothesis 1, no evidence of an association between medication non-adherence and suicide attempt was found. However, in the results of hypothesis 2, only the lowest medication adherence group had a higher risk of suicide attempt in our study, whereas the moderate medication adherence groups (0.2 ≤ PRM < 0.8) had reduced risks of suicide attempt. Differences in results may derive from discrepancies in study designs. The definition of medication adherence in the previous study was only categorized into binary variables (i.e., medication adherence and non-adherence), which may affect the sensitivity and specificity of the results. Patients were classified as nonadherent even if they had only one incidence of non-adherence over a 3-year follow-up period. Our characterization of adherence was more nuanced with a longer follow-up. Furthermore, we included the more general community patients rather than outpatients.

Lastly, about the results of NSSI, we found that medication non-adherence was associated with a higher risk of NSSI for hypothesis 1, while the findings were similar to that of suicide attempt for hypothesis 2. To our knowledge, this is the first study to examine the association between medication non-adherence and NSSI. Further research is needed to explore this problem in depth.

In summary, compared with the previous studies, we have the following strengths. First is the study design. The large sample size with a long follow-up period provided sufficient power to assess the associations of prolonged maintenance of antipsychotic medication with self-inflicted violence behaviors. The detailed classification of exposure adequately explored the association between medication non-adherence and different types of self-inflicted violence behaviors. Second, the analytic strategy. We employed causal inference techniques to identify and control for confounders. To guarantee the robustness of the results, comprehensive sensitivity analyses were conducted. In addition, we have taken into account inter-regional heterogeneity in analyses, which also improved the accuracy of the results.

### Potential mechanism

The present study shows patients with moderate or poor adherence (suicide: PRM < 0.8; suicide attempt and NSSI: 0.2 ≤ PRM < 0.8) had a lower incidence of self-inflicted violence behaviors. After performing a relevant literature review and consulting with psychiatrists, we suggested possible explanatory reasons as below: (1) in general, lack of knowledge and poor attitude towards the disease and antipsychotics contribute to medication non-adherence [23]. Antipsychotics are beneficial in reducing their symptoms and thus improving insight. Individuals with good medication adherence usually have enriched knowledge and better insight. “Insight paradox” could partly interpret our results: beneficial insight is paradoxical and is often associated with “post-psychotic depression,” where the patient’s insight is accompanied by feelings of shame and sadness, which may be due to stigmatization of mental illness, insufficient participation in mental health services, or low socioeconomic status [31]. As a result, patients might resort to self-inflicted violence behaviors [32]. (2) Antipsychotics also bring side effects such as extrapyramidal syndromes and metabolic and cardiovascular abnormalities [33, 34]. Good medication adherence accompanying adverse drug reactions likely contributes to elevated suicide risk [35].

Unlike the results of suicide, the risks of suicide attempt and NSSI were elevated in the lowest adherence groups in our study, which is possibly the result of different levels of suicidal intent of these behaviors. The strongest suicidality contributed to suicide completion. However, suicide attempt with nonviolent methods (e.g., cutting and poisoning) usually represents low suicidality and [36], as well as NSSI, serves as a maladaptive coping strategy for emotional dysregulation [37]. Similarly, patients dominated by severe symptoms (e.g., command hallucinations) might have low suicidal tendencies, which led them to engage in less harmful acts of self-inflicted violence behaviors (i.e., suicide attempt with nonviolent methods and NSSI). And the lowest medication adherence could lead to symptom development and emotional dysregulation.

However, since our dataset lacks these variables, we cannot build these potential mechanism pathways.

### Clinical implications

The results of this study warrant further investigation and public health action.

First, the classification of medication adherence should be further refined and assessed in community practice. In the present study, the results regarding suicide attempt and NSSI were inconsistent between the two- and five-classification of medication adherence. The reason for this is that the P1–P4 groups explored the medication non-adherence group (PRM < 0.8) in more detail. For suicide attempt, the lowest medication adherence group (P1) had an increased risk, while the moderate groups (P2–P4) showed a reduced risk compared with the control group (P5). These two associations in opposite directions in the P1 and the P2–P4 were combined generically in the analyses for hypothesis 1 into the medication non-adherence group, leading to the result that the association between medication non-adherence and suicide attempt was not statistically significant. We also found a similar phenomenon in the results of NSSI. As the associations were in the reverse direction for the P1 and P2–P4 groups, but stronger for the P1 group, it resulted in an elevated risk of NSSI in the medication non-adherence group. Therefore, further studies could investigate medication adherence in more in-depth and potential mediators to better help understand the underlying mechanisms of the above associations.

Second, the impact of medication non-adherence on different types of self-inflicted violence behaviors should be distinguished and emphasized in clinical and public health practice. Our findings showed that medication non-adherence did not appear to increase the risk of suicide. Likewise, patients with moderate adherence (0.2 ≤ PRM < 0.8) had a lower incidence of suicide attempt and NSSI. However, the lowest medication adherence was associated with a higher risk of suicide attempt and NSSI. Schizophrenia patients’ suicidal intent and involvement of psychological support, especially those with good medication adherence who might have been previously disregarded, need to be highlighted in community practices within middle- and low-income countries [38, 39].

### Limitations of this study

First, we captured medication adherence according to patient- or their family- reported, so possible concealment and forgetting might interfere with true medication use. The incidence of self-inflicted violence behaviors was lower in this study compared with relevant literatures [9, 40–42]. Besides the different target populations and follow-up durations across studies, this may also be due to a lack of objective confirmation of self-inflicted violence behaviors in this study. More objective indicators and measures are needed in the future.

Second, our definition of exposure led to an impossibility of differentiating the patient’s pattern of medication-taking (i.e., timing of the non-adherence event), e.g., if a patient is in long-term adherence in the early period but with poor medication adherence in the later period, the assignment of that patient to the adherence group is debatable.

Third, observational studies cannot avoid residual confounders. Although we used causal inference techniques such as DAG and inverse probability weighting to identify and control for confounders, and the *E*-value showed a moderate robustness of our results, causation cannot be inferred from the results. This study lacked the type of antipsychotic medication and mode of administration to support the exploration of possible mechanisms of drug effects. Future research could collect more comprehensive characteristics and possible time-varying confounders.

Finally, missing information existed in our database and we did not take any measure for missing values. Considering the relatively large sample size of this study, the participants with missing values were only a small fraction so we excluded them. Meanwhile, the distribution of most covariates was similar between individuals with missing values and included in the analyses (Additional file Table S28). This provides some assurance of the robustness of our results.

## Conclusions

In conclusion, our data found that medication adherence did not appear to reduce the risk of suicide. Likewise, patients with moderate adherence (0.2 ≤ PRM < 0.8) had a lower incidence of suicide and NSSI. However, almost no medication taken as prescribed (PRM < 0.2) was associated with an increased risk of suicide attempt and NSSI. These findings suggest the need for more detailed portrait of medication adherence as well as accessible and high-quality integrated mental health care in the community to prevent self-inflicted violence behaviors in middle- and low-income countries.

### Supplementary Information

**Additional file 1:** Supplementary Method. **Figure S1.** Screening flow chart. **Figure S2.** Directed acyclic graphs. **Table S1.** Definition and encoding of variables in this study. **Table S2.** Group differences of the incidence of self-inflicted violence behaviors during follow-up. **Table S3.** Reginal differences of the incidence of self-inflicted violence behaviors during follow-up. **Table S4.** Characteristics for the suicide attempt cohort of participants (for hypothesis 1). **Table S5.** Characteristics for the NSSI cohort of participants (for hypothesis 1). **Table S6.** Characteristics for the weighting suicide cohort of participants (for hypothesis 2). **Table S7.** Characteristics for the weighting suicide attempt cohort of participants (for hypothesis 2). **Table S8.** Characteristics for the weighting NSSI cohort of participants (for hypothesis 2). **Table S9.** Association between 2 levels of medication adherence and self-inflicted violence behaviors (follow-up of ≥1 year for hypothesis 1). **Table S10.** Association between 5 levels of medication adherence and self-inflicted violence behaviors (follow-up of ≥1 year for hypothesis 2). **Table S11.** Association between 2 levels of medication adherence and self-inflicted violence behaviors (the number of records of medication adherence ≥ 5 for hypothesis 1). **Table S12.** Association between 5 levels of medication adherence and self-inflicted violence behaviors (the number of records of medication adherence ≥ 5 for hypothesis 2). **Table S13.** Association between 2 levels of medication adherence and self-inflicted violence behaviors (Re-define the PRM for hypothesis 1). **Table S14.** Association between 5 levels of medication adherence and self-inflicted violence behaviors (Re-define the PRM for hypothesis 2). **Table S15.** E-value for quantifying unmeasured confounders. **Table S16.** Sex-stratified association between 2 levels of medication adherence and self-inflicted violence behaviors during follow-up period (for hypothesis 1). **Table S17.** Association between 5 levels of medication adherence and self-inflicted violence behaviors among male patients (for hypothesis 2). **Table S18.** Association between 5 levels of medication adherence and self-inflicted violence behaviors among female patients (for hypothesis 2). **Table S19.** Urbanity-stratified association between 2 levels of medication adherence and self-inflicted violence behaviors during follow-up period (for hypothesis 1). **Table S20.** Association between 5 levels of medication adherence and self-inflicted violence behaviors among rural patients (for hypothesis 2). **Table S21.** Association between 5 levels of medication adherence and self-inflicted violence behaviors among urban patients (for hypothesis 2). **Table S22.** Age-stratified association between 2 levels of medication adherence and self-inflicted violence behaviors during follow-up period (for hypothesis 1). **Table S23.** Association between 5 levels of medication adherence and self-inflicted violence behaviors among patients aged 10 to 24 (for hypothesis 2). **Table S24.** Association between 5 levels of medication adherence and self-inflicted violence behaviors among patients aged 25 to 44 (for hypothesis 2). **Table S25.** Association between 5 levels of medication adherence and self-inflicted violence behaviors among patients aged 45 to 59 (for hypothesis 2). **Table S26.** Association between 5 levels of medication adherence and self-inflicted violence behaviors among patients aged ≥60 (for hypothesis 2). **Table S27.** Non-proportional hazards test for main and subgroup analyses. **Table S28.** Comparison of characteristics between the individuals with missing values and the included sample.

## Abbreviations

CI: Confidence interval MPR Medication possession ratio IPW Inverse probability weighting
DAG: Directed acyclic graph
HR: Hazard ratio
ICD-10: The International Statistical Classification of Diseases and Related Health Problems 10th Revision STROBE The Strengthening the Reporting of Observational Studies in Epidemiology
NSSI: Non-suicidal self-injury
PRM: The proportion of regular medication
SD: Standard deviation SMD Standardized mean difference
