# Supplementary material for: Medication non-adherence and self-inflicted violence behaviors among 185,800 patients with schizophrenia in the community: a 12-year cohort study
Source: BMC Med. 2024 Mar 25;22:136. doi: 10.1186/s12916-024-03354-7 (PMC10962179; doi:10.1186/s12916-024-03354-7)
Supplement: Supplementary file 1 — Additional file 1: Supplementary Method. Figure S1. Screening flow chart. Figure S2. Directed acyclic graphs. Table S1. Definition and encoding of variables in this study. Table S2. Group differences of the incidence of self-inflicted violence behaviors during follow-up. Table S3. Reginal differences of the incidence of self-inflicted violence behaviors during follow-up. Table S4. Characteristics for the suicide attempt cohort of participants (for hypothesis 1). Table S5. Characteristics for the NSSI cohort of participants (for hypothesis 1). Table S6. Characteristics for the weighting suicide cohort of participants (for hypothesis 2). Table S7. Characteristics for the weighting suicide attempt cohort of participants (for hypothesis 2). Table S8. Characteristics for the weighting NSSI cohort of participants (for hypothesis 2). Table S9. Association between 2 levels of medication adherence and self-inflicted violence behaviors (follow-up of ≥1 year for hypothesis 1). Table S10. Association between 5 levels of medication adherence and self-inflicted violence behaviors (follow-up of ≥1 year for hypothesis 2). Table S11. Association between 2 levels of medication adherence and self-inflicted violence behaviors (the number of records of medication adherence ≥ 5 for hypothesis 1). Table S12. Association between 5 levels of medication adherence and self-inflicted violence behaviors (the number of records of medication adherence ≥ 5 for hypothesis 2). Table S13. Association between 2 levels of medication adherence and self-inflicted violence behaviors (Re-define the PRM for hypothesis 1). Table S14. Association between 5 levels of medication adherence and self-inflicted violence behaviors (Re-define the PRM for hypothesis 2). Table S15. E-value for quantifying unmeasured confounders. Table S16. Sex-stratified association between 2 levels of medication adherence and self-inflicted violence behaviors during follow-up period (for hypothesis 1). Table S17. Association between 5 [file 12916_2024_3354_MOESM1_ESM.pdf]

Medication non-adherence and the risk of self-inflicted violence behaviors among 185,800 patients with schizophrenia in the community: a 12-year cohort study

Additional File

Supplementary Method

Supplementary Figures

Figure S1. Screening flow chart

Figure S2. Directed acyclic graphs

Supplementary Tables

Table S1. Definition and encoding of variables in this study

Table S2. Group differences of the incidence of self-inflicted violence behaviors during follow-up

Table S3. Reginal differences of the incidence of self-inflicted violence behaviors during follow-up

Table S4. Characteristics for the suicide attempt cohort of participants (for hypothesis 1)

Table S5. Characteristics for the NSSI cohort of participants (for hypothesis 1)

Table S6. Characteristics for the weighting suicide cohort of participants (for hypothesis 2)

Table S7. Characteristics for the weighting suicide attempt cohort of participants (for hypothesis 2)

Table S8. Characteristics for the weighting NSSI cohort of participants (for hypothesis 2)

Table S9. Association between 2 levels of medication adherence and self-inflicted violence behaviors (follow-up of ≥1 year for hypothesis 1)

Table S10. Association between 5 levels of medication adherence and self-inflicted violence behaviors (follow-up of ≥1 year for hypothesis 2)

Table S11. Association between 2 levels of medication adherence and self-inflicted violence behaviors (the number of records of medication adherence ≥ 5 for hypothesis 1)
Table S12. Association between 5 levels of medication adherence and self-inflicted violence behaviors (the number of records of medication adherence ≥ 5 for hypothesis 2)
Table S13. Association between 2 levels of medication adherence and self-inflicted violence behaviors (Re-define the PRM for hypothesis 1)
Table S14. Association between 5 levels of medication adherence and self-inflicted violence behaviors (Re-define the PRM for hypothesis 2)
Table S15. E-value for quantifying unmeasured confounders
Table S16. Sex-stratified association between 2 levels of medication adherence and self-inflicted violence behaviors during follow-up period (for hypothesis 1)
Table S17. Association between 5 levels of medication adherence and self-inflicted violence behaviors among male patients (for hypothesis 2)
Table S18. Association between 5 levels of medication adherence and self-inflicted violence behaviors among female patients (for hypothesis 2)
Table S19. Urbanity-stratified association between 2 levels of medication adherence and self-inflicted violence behaviors during follow-up period (for hypothesis 1)
Table S20. Association between 5 levels of medication adherence and self-inflicted violence behaviors among rural patients (for hypothesis 2)
Table S21. Association between 5 levels of medication adherence and self-inflicted violence behaviors among urban patients (for hypothesis 2)
Table S22. Age-stratified association between 2 levels of medication adherence and self-inflicted violence behaviors during follow-up period (for hypothesis 1)
Table S23. Association between 5 levels of medication adherence and self-inflicted violence behaviors among patients aged 10 to 24 (for hypothesis 2)
Table S24. Association between 5 levels of medication adherence and self-inflicted violence behaviors among patients aged 25 to 44 (for hypothesis 2)
Table S25. Association between 5 levels of medication adherence and self-inflicted violence behaviors among patients aged 45 to 59 (for hypothesis 2)
Table S26. Association between 5 levels of medication adherence and self-inflicted violence behaviors among patients aged ≥60 (for hypothesis 2)
Table S27. Non-proportional hazards test for main and subgroup analyses
Table S28. Comparison of characteristics between the individuals with missing values and the included sample

Supplementary Method

Non-linear association

We used Cox proportional hazards model to estimate time-to-event associations of PRM and the risk of self-inflicted violence behaviors during follow-up. We utilized linear models to estimate stabilized weights for PRM. To assess balance in the distributions of covariates for continuous exposure within weighted cohorts, we examined the Pearson correlation between each covariate and PRM. A correlation of 0.1 or lower was deemed indicative of ideal balance. To evaluate potential non-linear associations, dose-response relationships were assessed by restricted cubic spline function with 4 knots[1]. Non-linear associations were tested using the Wald statistics. The analysis was done using the R package "rms".

Reference

1. Desquilbet L, Mariotti F. Dose-response analyses using restricted cubic spline functions in public health research. Stat Med. 2010;29(9):1037-57.1.

Supplementary Figures

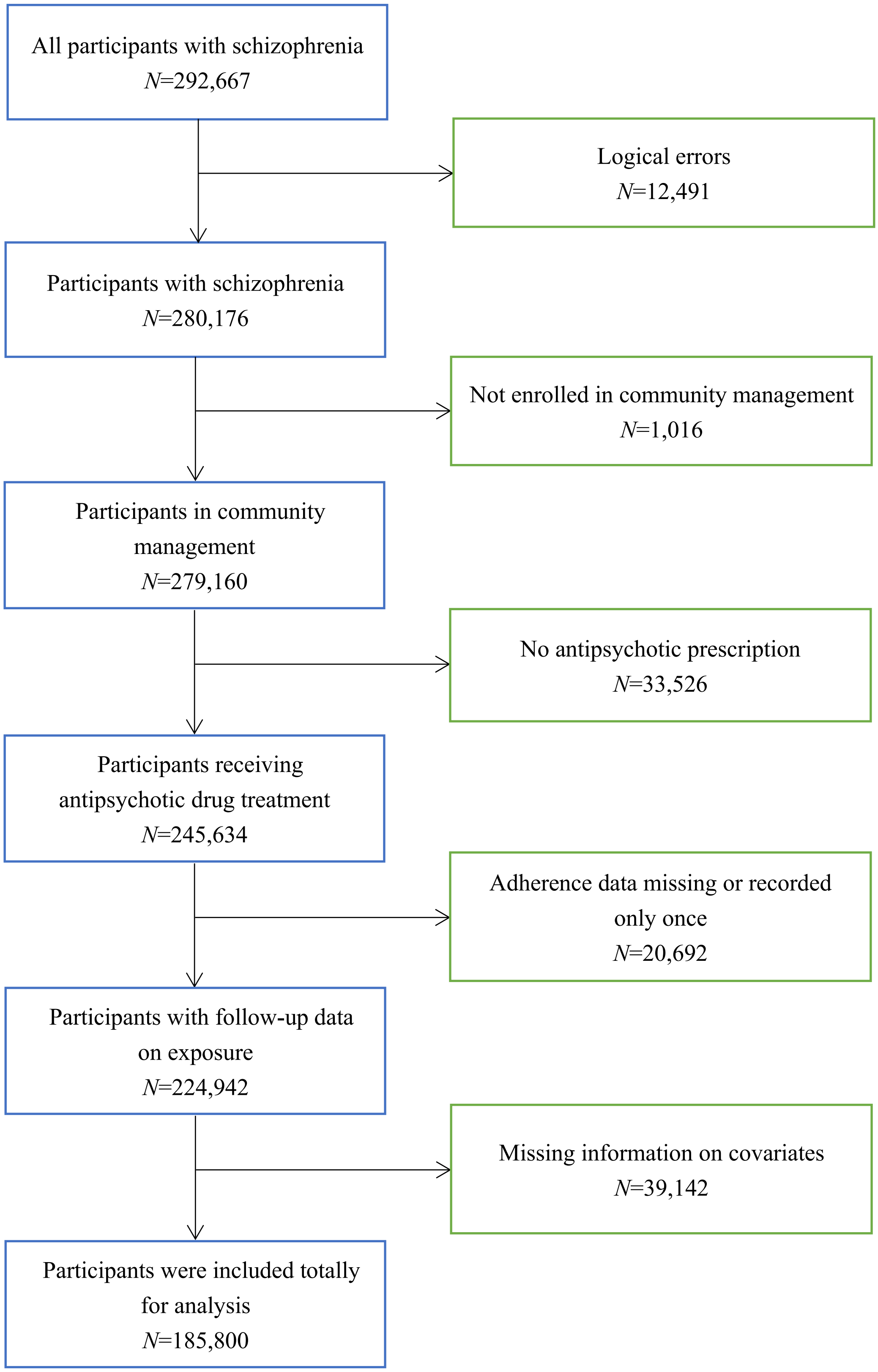

Figure S1. Screening flow chart

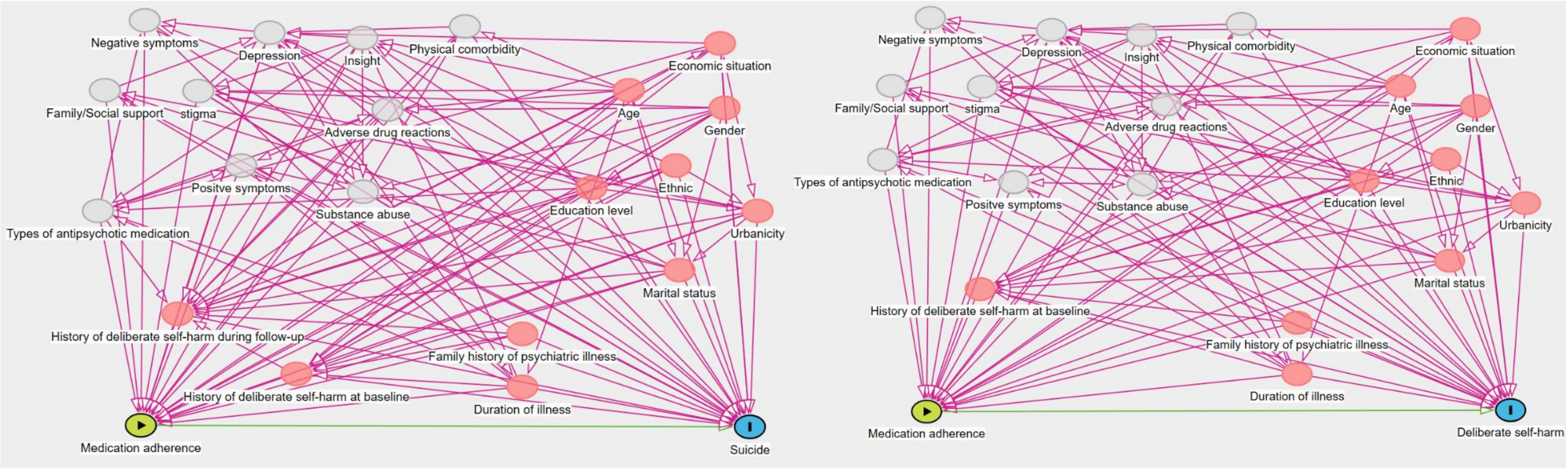

Figure S2. Directed acyclic graphs

The yellow and blue nodes indicate exposure and outcomes, respectively. The pink and grey nodes are observed and unmeasured confounders, respectively. Deliberate self-harm contains suicide attempt and NSSI.

Supplementary Tables

Table S1. Definition and encoding of variables in this study

Variable type	Name	Definition
**Outcomes**	Suicide attempt	Nonfatal, self-directed, potentially injurious behavior with an intent to die as a result of the behavior, even if the behavior does not result in injury.<br>Non-occurrence=0; Occurrence=1
	Nonsuicidal self-injury	Intentional self-injury behavior without intent to die, distinguish the behavior from suicide attempt.<br>Non-occurrence=0; Occurrence=1
	Suicide	Death caused by self-directed injurious behavior with an intent to die as a result of the behavior.<br>Non-occurrence=0; Occurrence=1
	Self-inflicted violence behaviors	Violence against self among patients with schizophrenia includes suicide attempt, nonsuicidal self-injury, and suicide.
**Exposure**	Medication non-adherence	PRM: the proportion of "regular adherence" days to the total number of days with antipsychotic treatment from the initial follow-up to the event of interest or censoring. Please see the details in "Exposure" in the main body of this paper.<br>Binary for hypothesis 1:<br>Non-adherence=0 (PRM≥0.8);<br>Non-adherence=1 (PRM<0.8)<br>Five classifications for hypothesis 2:<br>{P5=0 (PRM≥0.8) ***VS*** P4=1 (0.6≤PRM<0.8)};<br>{P5=0 (PRM≥0.8) ***VS*** P3=1 (0.40≤PRM<0.6)};<br>{P5=0 (PRM≥0.8) ***VS*** P2=1 (0.20≤PRM<0.4)};<br>{P5=0 (PRM≥0.8) ***VS*** P1=1 (0≤PRM<0.2)}
**Confounders**	Age, years	Age at baseline
	Sex	Female=0; Male=1

Ethnic	Minorities=0; Han=1
Family history of psychiatric illness	Family history of severe psychiatric illness in two or three generations:<br>None = 0; Yes = 1
Marital status	Never married = 1; Married= 2; Widowed =3; Divorced =4
Urbanicity	Urban=0; Rural=1
Education level	The highest level of education obtained by the patient at baseline is divided into 3 levels:<br>Primary school or lower=1; Middle and high school=2; College or university or higher=3
Economic situation	Poverty refers to the economic situation that is below the local poverty line by income level:<br>Non-poverty=0; Poverty=1
Duration of illness, years	Time of symptom onset at baseline:<br><10 years=1; 10-20=2; 20-30=3; ≥30=4
History of suicide attempt at baseline	At baseline, patients and their family were asked about any history of suicide attempt<br>No = 0; Yes = 1; Unknown =2;
History of NSSI at baseline	At baseline, patients and their family were asked about any history of NSSI<br>No = 0; Yes = 1; Unknown =2;
History of suicide attempt during follow-up	Suicide attempt during follow-up<br>No = 0; Yes = 1;
History of NSSI during follow-up	NSSI during follow-up<br>No = 0; Yes = 1;
History of self-harm	In the suicide cohort, a history of self-harm was defined as a history of suicide attempt or NSSI at baseline or during follow-up; in the suicide attempt and NSSI cohort, a history of self-harm included a history of suicide attempt or NSSI at baseline.<br>No = 0; Yes = 1.

Table S2. Group differences of the incidence of self-inflicted violence behaviors during follow-up

Self-inflicted violence behaviors	Medication adherence	Medication non-adherence					*P* value †
	P5	P4	P3	P2	P1	Total	
Before weighting							
Suicide	259(0.4)	51(0.3)	45(0.2)	58(0.3)	160(0.3)	314(0.3)	<0.001
Suicide attempt	352(0.6)	60(0.3)	73(0.4)	82(0.4)	545(0.9)	760(0.6)	0.058
NSSI	344(0.5)	93(0.5)	79(0.4)	118(0.5)	758(1.2)	1,048(0.9)	<0.001
After weighting							
Suicide	251.6(0.4)	48.8(0.2)	45.1(0.2)	65.0(0.3)	187.8(0.3)	334.9(0.3)	<0.001
Suicide attempt	346.6(0.5)	61.9(0.3)	75.5(0.4)	81.0(0.4)	609.4(1.0)	781.9(0.6)	0.007
NSSI	361.1(0.6)	95.0(0.5)	78.9(0.4)	118.4(0.5)	756.3(1.2)	1,027.9(0.8)	<0.001

Results are expressed as frequency and incidence in each group.

† χ^2 tests were conducted between medication adherence group and medication non-adherence group.

Table S3. Reginal differences of the incidence of self-inflicted violence behaviors during follow-up

Region	n	Suicide	Suicide attempt	NSSI
1	32,887	92(0.3)	164(0.5)	153(0.5)
2	10,204	41(0.4)	60(0.6)	54(0.5)
3	2,424	7(0.3)	12(0.5)	14(0.6)
4	10,791	31(0.3)	52(0.5)	66(0.6)
5	7,491	31(0.4)	39(0.5)	45(0.6)
6	11,307	61(0.5)	62(0.5)	70(0.6)
7	5,700	24(0.4)	58(1.0)	67(1.2)
8	6,844	15(0.2)	32(0.5)	32(0.5)
9	11,689	42(0.4)	90(0.8)	93(0.8)
10	5,150	17(0.3)	49(1.0)	44(0.9)
11	14,595	33(0.2)	102(0.7)	171(1.2)
12	7,367	22(0.3)	22(0.3)	30(0.4)
13	11,014	34(0.3)	49(0.4)	47(0.4)
14	9,192	28(0.3)	48(0.5)	49(0.5)
15	14,907	38(0.3)	73(0.5)	124(0.8)
16	2,459	12(0.5)	26(1.1)	23(0.9)
17	9,084	19(0.2)	106(1.2)	208(2.3)
18	9,867	21(0.2)	48(0.5)	50(0.5)
19	404	1(0.2)	3(0.7)	6(1.5)
20	147	0(0.0)	2(1.4)	1(0.7)
21	2,276	4(0.2)	15(0.7)	45(2.0)
***P* value**	-	0.001	<0.001	<0.001

Table S4. Characteristics for the suicide attempt cohort of participants (for hypothesis 1)

	Unweighted				IPW†			
	Medication adherence (n = 63,899)	Medication non-adherence (n = 121,901)	*P* value	SMD	Medication adherence (n = 63,669.01)	Medication non-adherence (n = 122,225.73)	*P* value	SMD
**Sociodemographic characteristics**								
Age, mean (SD), y	44.70(14.33)	48.96(14.46)	<0.001	0.296	47.33(14.47)	47.37(14.64)	0.663	0.002
Sex			<0.001	0.026			0.657	0.002
Male	30,181(47.2)	55,977(45.9)			29,426.4(46.2)	56,633.3(46.3)		
Female	33,718(52.8)	65,924(54.1)			34,242.6(53.8)	65,592.5(53.7)		
Ethnic			0.822	0.001			0.860	0.001
Han	63,335(99.1)	120,839(99.1)			63,089.5(99.1)	121,124.0(99.1)		
Minorities	564(0.9)	1,062(0.9)			579.6(0.9)	1,101.7(0.9)		
Marital status			<0.001	0.169			<0.001	0.055
Never married	19,062(29.8)	32,095(26.3)			16,647.9(26.1)	33,707.8(27.6)		
Married	37,952(59.4)	77,604(63.7)			40,870.8(64.2)	75,605.6(61.9)		
Widowed	2,226(3.5)	6,631(5.4)			2,657.6(4.2)	6,051.1(5.0)		
Divorced	4,659(7.3)	5,571(4.6)			3,492.7(5.5)	6,861.2(5.6)		
Urbanicity			<0.001	0.596			0.487	0.004
Rural	41,786(65.4)	108,845(89.3)			51,551.1(81.0)	98,793.0(80.8)		
Urban	22,113(34.6)	13,056(10.7)			12,118.0(19.0)	23,432.7(19.2)		
Education level			<0.001	0.512			<0.001	0.028
Primary school or lower	32,727(51.2)	90,805(74.5)			42,254.4(66.4)	81,279.2(66.5)		
Middle and high school	28,464(44.5)	30,062(24.7)			20,183.5(31.7)	38,110.8(31.2)		
College/university or higher	2,708(4.2)	1,034(0.8)			1,231.1(1.9)	2,835.8(2.3)		
Economic situation			<0.001	0.300			0.933	<0.001

Poverty	35,273(55.2)	84,829(69.6)			41,080.8(64.5)	78,837.6(64.5)		
Non-poverty	28,626(44.8)	37,072(30.4)			22,588.2(35.5)	43,388.1(35.5)		
**Clinical characteristics**								
Family history of psychiatric illness			<0.001	0.061			0.965	<0.001
Yes	3,554(5.6)	5,183(4.3)			2,984.9(4.7)	5,724.3(4.7)		
No	60,345(94.4)	116,718(95.7)			60,684.1(95.3)	116,501.4(95.3)		
Duration of illness								
mean (SD), years	11.60(10.42)	13.40(11.49)	<0.001	0.163	12.39(11.08)	12.66(11.14)	<0.001	0.024
<10	34,446(53.9)	58,173(47.7)	<0.001	0.158	32,529.8(51.1)	61,766.7(50.5)	0.072	0.014
10~19	16,601(26.0)	32,390(26.6)			16,507.8(25.9)	32,006.8(26.2)		
20~29	8,543(13.4)	18,946(15.5)			9,207.8(14.5)	17,648.0(14.4)		
≥30	4,309(6.7)	12,392(10.2)			5,423.6(8.5)	10,804.3(8.8)		
**History of self-inflicted violence behaviors**								
History of suicide attempt at baseline			<0.001	0.032				
Yes	280(0.4)	571(0.5)			262.6(0.4)	601.8(0.5)	0.071	0.012
No	58,843(92.1)	113,225(92.9)			58,944.1(92.6)	113,146.4(92.6)		
Unknown	4,776(7.5)	8,105(6.6)			4,462.4(7.0)	8,477.5(6.9)		
History of NSSI at baseline			<0.001	0.038			0.042	0.014
Yes	208(0.3)	601(0.5)			239.6(0.4)	566.3(0.5)		
No	58,736(91.9)	112,735(92.5)			58,739.9(92.3)	112,760.2(92.3)		
Unknown	4,955(7.8)	8,565(7.0)			4,689.5(7.4)	8,899.3(7.3)		

† Inverse probability weighting

Table S5. Characteristics for the NSSI cohort of participants (for hypothesis 1)

	Unweighted				IPW†			
	Medication adherence (n = 63,904)	Medication non-adherence (n = 121,896)	*P* value	SMD	Medication adherence (n = 63,676.74)	Medication non-adherence (n = 122,217.14)	*P* value	SMD
**Sociodemographic characteristics**								
Age, mean (SD), y	44.70(14.33)	48.95(14.46)	<0.001	0.295	47.33(14.47)	47.37(14.63)	0.678	0.002
Sex			<0.001	0.026			0.648	0.002
Male	30,187(47.2)	55,971(45.9)			29,425.7(46.2)	56,625.0(46.3)		
Female	33,717(52.8)	65,925(54.1)			34,251.1(53.8)	65,592.1(53.7)		
Ethnic			0.743	0.002			0.869	0.001
Han	63,338(99.1)	120,836(99.1)			63,097.2(99.1)	121,115.0(99.1)		
Minorities	566(0.9)	1,060(0.9)			579.5(0.9)	1,102.1(0.9)		
Marital status			<0.001	0.169			<0.001	0.055
Never married	19,063(29.8)	32,094(26.3)			16,650.6(26.1)	33,701.4(27.6)		
Married	37,954(59.4)	77,602(63.7)			40,877.8(64.2)	75,607.5(61.9)		
Widowed	2,225(3.5)	6,632(5.4)			2,655.9(4.2)	6,053.5(5.0)		
Divorced	4,662(7.3)	5,568(4.6)			3,492.5(5.5)	6,854.8(5.6)		
Urbanicity			<0.001	0.596			0.491	0.004
Rural	41,786(65.4)	108,845(89.3)			51,558.9(81.0)	98,790.7(80.8)		
Urban	22,118(34.6)	13,051(10.7)			12,117.8(19.0)	23,426.4(19.2)		
Education level			<0.001	0.512			<0.001	0.029
Primary school or lower	32,728(51.2)	90,804(74.5)			42,259.0(66.4)	81,279.0(66.5)		
Middle and high school	28,470(44.6)	30,056(24.7)			20,187.7(31.7)	38,097.0(31.2)		
College/university or higher	2,706(4.2)	1,036(0.8)			1,230.0(1.9)	2,841.1(2.3)		
Economic situation			<0.001	0.301			0.932	<0.001

Poverty	35,270(55.2)	84,832(69.6)			41,086.9(64.5)	78,833.7(64.5)		
Non-poverty	28,634(44.8)	37,064(30.4)			22,589.8(35.5)	43,383.5(35.5)		
**Clinical characteristics**								
Family history of psychiatric illness			<0.001	0.061			0.961	<0.001
Yes	3,555(5.6)	5,182(4.3)			2,983.8(4.7)	5,720.4(4.7)		
No	60,349(94.4)	116,714(95.7)			60,693.0(95.3)	116,496.8(95.3)		
Duration of illness								
mean (SD), years	11.60(10.42)	13.40(11.49)	<0.001	0.163	12.39(11.08)	12.66(11.14)	<0.001	0.024
<10	34,447(53.9)	58,172(47.7)	<0.001	0.158	32,532.7(51.1)	61,762.6(50.5)	0.076	0.014
10~19	16,606(26.0)	32,385(26.6)			16,511.2(25.9)	32,000.7(26.2)		
20~29	8,541(13.4)	18,948(15.5)			9,206.6(14.5)	17,649.3(14.4)		
≥30	4,310(6.7)	12,391(10.2)			5,426.3(8.5)	10,804.5(8.8)		
**History of self-inflicted violence behaviors**								
History of suicide attempt at baseline			<0.001	0.034			0.012	0.016
Yes	269(0.4)	582(0.5)			249.7(0.4)	605.9(0.5)		
No	58,853(92.1)	113,215(92.9)			58,960.3(92.6)	113,141.2(92.6)		
Unknown	4,782(7.5)	8,099(6.6)			4,466.8(7.0)	8,470.1(6.9)		
History of NSSI at baseline			<0.001	0.036			0.100	0.012
Yes	219(0.3)	590(0.5)			246.9(0.4)	563.7(0.5)		
No	58,722(91.9)	112,749(92.5)			58,746.2(92.3)	112,755.5(92.3)		
Unknown	4,963(7.8)	8,557(7.0)			4,683.6(7.4)	8,897.9(7.3)		

† Inverse probability weighting

Table S6. Characteristics for the weighting suicide cohort of participants (for hypothesis 2)

	P5 (n = 63,728.65)	P4 (n = 19,913.1)	P3 (n = 19,471.85)	P2 (n = 21,786.34)	P1 (n = 61,202.5)	SMD
**Sociodemographic characteristics**						
Age, mean (SD), y	47.41(14.53)	47.46(14.47)	47.48(14.42)	47.36(14.48)	47.25(14.77)	0.008
Sex						0.007
Male	29,525.8(46.3)	9,241.9(46.4)	9,034.3(46.4)	10,195.8(46.8)	28,126.4(46.0)	
Female	34,202.8(53.7)	10,671.2(53.6)	10,437.6(53.6)	11,590.5(53.2)	33,076.1(54.0)	
Ethnic						0.002
Han	63,141.0(99.1)	19,735.0(99.1)	19,297.0(99.1)	21,586.5(99.1)	60,633.6(99.1)	
Minorities	587.7(0.9)	178.1(0.9)	174.8(0.9)	199.8(0.9)	568.9(0.9)	
Marital status						0.048
Never married	16,662.5(26.1)	5,158.0(25.9)	5,138.5(26.4)	5,700.0(26.2)	17,455.4(28.5)	
Married	40,887.9(64.2)	12,899.3(64.8)	12,470.4(64.0)	14,002.3(64.3)	36,646.6(59.9)	
Widowed	2,682.2(4.2)	851.3(4.3)	839.0(4.3)	968.2(4.4)	3,328.3(5.4)	
Divorced	3,496.0(5.5)	1,004.5(5.0)	1,023.9(5.3)	1,115.8(5.1)	3,772.2(6.2)	
Urbanicity						0.007
Rural	51,647.2(81.0)	16,146.8(81.1)	15,793.2(81.1)	17,624.3(80.9)	49,285.0(80.5)	
Urban	12,081.4(19.0)	3,766.3(18.9)	3,678.7(18.9)	4,162.0(19.1)	11,917.5(19.5)	
Education level						0.014
Primary school or lower	42,407.5(66.5)	13,248.7(66.5)	12,971.5(66.6)	14,496.4(66.5)	40,665.9(66.4)	
Middle and high school	20,078.0(31.5)	6,244.7(31.4)	6,067.5(31.2)	6,817.3(31.3)	19,074.7(31.2)	
College/university or	1,243.1(2.0)	419.7(2.1)	432.9(2.2)	472.6(2.2)	1,462.0(2.4)	

higher						
Economic situation						0.004
Poverty †	41,162.8(64.6)	12,865.0(64.6)	12,600.2(64.7)	14,046.0(64.5)	39,317.0(64.2)	
Non-poverty	22,565.9(35.4)	7,048.1(35.4)	6,871.7(35.3)	7,740.4(35.5)	21,885.5(35.8)	
**Clinical characteristics**						
Family history of psychiatric illness						0.005
Yes	2,986.2(4.7)	941.6(4.7)	890.2(4.6)	1,010.8(4.6)	2,928.4(4.8)	
No	60,742.4(95.3)	18,971.5(95.3)	18,581.7(95.4)	20,775.6(95.4)	58,274.1(95.2)	
Duration of illness						
mean (SD), years	12.45(11.14)	12.69(11.16)	12.71(11.17)	12.60(11.24)	12.62(11.04)	0.011
<10	32,510.7(51.0)	9,934.5(49.9)	9,754.0(50.1)	11,134.7(51.1)	31,097.7(50.8)	0.02
10~19	16,456.0(25.8)	5,256.4(26.4)	5,115.9(26.3)	5,599.4(25.7)	16,042.3(26.2)	
20~29	9,217.4(14.5)	2,970.5(14.9)	2,873.5(14.8)	3,047.5(14.0)	8,767.0(14.3)	
≥30	5,544.5(8.7)	1,751.7(8.8)	1,728.4(8.9)	2,004.7(9.2)	5,295.5(8.7)	
**History of self-inflicted violence behaviors**						
History of suicide attempt at baseline						0.012
Yes	253.2(0.4)	100.3(0.5)	108.2(0.6)	113.3(0.5)	270.6(0.4)	
No	59,011.7(92.6)	18,419.1(92.5)	18,016.4(92.5)	20,162.8(92.5)	566,91.8(92.6)	
Unknown	4,463.7(7.0)	1,393.7(7.0)	1,347.2(6.9)	1,510.2(6.9)	4,240.1(6.9)	
History of NSSI at baseline						0.009

Yes	240.3(0.4)	93.0(0.5)	95.0(0.5)	103.0(0.5)	260.7(0.4)	
No	58,808.5(92.3)	18,355.2(92.2)	17,960.3(92.2)	20,097.7(92.2)	56,500.7(92.3)	
Unknown	4,679.9(7.3)	1,465.0(7.4)	1,416.6(7.3)	1,585.7(7.3)	4,441.1(7.3)	
History of suicide attempt during follow-up						0.002
Yes	386.8(0.6)	120.7(0.6)	118.7(0.6)	127.7(0.6)	355.3(0.6)	
No	63,341.8(99.4)	19,792.4(99.4)	19,353.1(99.4)	21,658.7(99.4)	60,847.2(99.4)	
History of NSSI during follow-up						0.003
Yes	461.6(0.7)	154.0(0.8)	146.8(0.8)	164.2(0.8)	443.7(0.7)	
No	63,267.0(99.3)	19,759.2(99.2)	19,325.1(99.2)	21,622.2(99.2)	60,758.8(99.3)	

Table S7. Characteristics for the weighting suicide attempt cohort of participants (for hypothesis 2)

	P5 (n = 63,770.08)	P4 (n = 19,815.47)	P3 (n = 19,409.92)	P2 (n = 21,723.39)	P1 (n = 61,395.12)	SMD
**Sociodemographic characteristics**						
Age, mean (SD), y	47.41(14.53)	47.46(14.47)	47.47(14.42)	47.37(14.48)	47.24(14.77)	0.008
Sex						0.007
Male	29,541.7(46.3)	9,194.1(46.4)	9,008.2(46.4)	10,170.5(46.8)	28,205.3(45.9)	
Female	34,228.4(53.7)	10,621.4(53.6)	10,401.8(53.6)	11,552.9(53.2)	33,189.8(54.1)	
Ethnic						0.002
Han	63,183.1(99.1)	19,638.3(99.1)	19,235.4(99.1)	21,523.4(99.1)	60,825.9(99.1)	
Minorities	587.0(0.9)	177.2(0.9)	174.5(0.9)	200.0(0.9)	569.2(0.9)	
Marital status						0.048
Never married	16,672.6(26.1)	5,135.6(25.9)	5,127.7(26.4)	5,678.8(26.1)	17,518.0(28.5)	
Married	40,917.8(64.2)	12,830.9(64.8)	12,425.4(64.0)	13,966.6(64.3)	36,756.3(59.9)	
Widowed	2,682.1(4.2)	849.5(4.3)	833.9(4.3)	965.9(4.4)	3,332.3(5.4)	
Divorced	3,497.6(5.5)	999.5(5.0)	1,022.8(5.3)	1,112.1(5.1)	3,788.5(6.2)	
Urbanicity						0.007
Rural	51,682.2(81.0)	16,066.8(81.1)	15,741.7(81.1)	17,576.2(80.9)	49,429.8(80.5)	
Urban	12,087.9(19.0)	3,748.7(18.9)	3,668.2(18.9)	4,147.2(19.1)	11,965.3(19.5)	
Education level						0.014
Primary school or lower	42,433.9(66.5)	13,181.1(66.5)	12,931.0(66.6)	14,447.8(66.5)	40,785.4(66.4)	
Middle and high school	20,092.4(31.5)	6,218.1(31.4)	6,042.4(31.1)	6,807.1(31.3)	19,147.0(31.2)	
College/university or	1,243.8(2.0)	416.3(2.1)	436.5(2.2)	468.6(2.2)	1,462.7(2.4)	

higher						
Economic situation						0.004
Poverty †	41,193.5(64.6)	12,803.7(64.6)	12,557.1(64.7)	14,001.2(64.5)	39,447.7(64.3)	
Non-poverty	22,576.6(35.4)	7,011.8(35.4)	6,852.8(35.3)	7,722.2(35.5)	21,947.4(35.7)	
**Clinical characteristics**						
Family history of psychiatric illness						0.005
Yes	2,986.2(4.7)	936.1(4.7)	888.7(4.6)	1,009.1(4.6)	2,936.0(4.8)	
No	60,783.9(95.3)	18,879.4(95.3)	18,521.2(95.4)	20,714.3(95.4)	58,459.1(95.2)	
Duration of illness						
mean (SD), years	12.45(11.14)	12.68(11.16)	12.71(11.17)	12.60(11.24)	12.62(11.04)	0.011
<10	32,532.6(51.0)	9,888.1(49.9)	9,726.9(50.1)	11,090.3(51.1)	31,196.9(50.8)	0.02
10~19	16,463.8(25.8)	5,226.2(26.4)	5,095.0(26.2)	5,603.1(25.8)	16,094.5(26.2)	
20~29	9,224.1(14.5)	2,958.4(14.9)	2,864.2(14.8)	3,031.9(14.0)	8,797.6(14.3)	
≥30	5,549.6(8.7)	1,742.7(8.8)	1,723.7(8.9)	1,998.1(9.2)	5,306.2(8.6)	
**History of self-inflicted violence behaviors**						
History of suicide attempt at baseline						0.01
Yes	263.5(0.4)	85.5(0.4)	89.9(0.5)	106.4(0.5)	333.4(0.5)	
No	59,039.4(92.6)	18,336.7(92.5)	17,969.2(92.6)	20,106.6(92.6)	56,827.5(92.6)	
Unknown	4,467.2(7.0)	1,393.3(7.0)	1,350.9(7.0)	1,510.4(7.0)	4,234.2(6.9)	
History of NSSI at baseline						0.008

Yes	240.4(0.4)	91.8(0.5)	93.7(0.5)	100.8(0.5)	276.3(0.5)
No	58,835.7(92.3)	18,265.7(92.2)	17,903.5(92.2)	20,039.6(92.2)	56,655.8(92.3)
Unknown	4,694.0(7.4)	1,458.0(7.4)	1,412.7(7.3)	1,583.0(7.3)	4,463.0(7.3)

Table S8. Characteristics for the weighting NSSI cohort of participants (for hypothesis 2)

	P5 (n = 63,779.01)	P4 (n = 19,823.34)	P3 (n = 19,353.57)	P2 (n = 21,658.15)	P1 (n = 61,493.83)	SMD
**Sociodemographic characteristics**						
Age, mean (SD), y	47.41(14.53)	47.46(14.47)	47.48(14.41)	47.36(14.48)	47.25(14.77)	0.008
Sex						0.007
Male	29,542.3(46.3)	9,198.1(46.4)	8,980.0(46.4)	10,142.4(46.8)	28,249.8(45.9)	
Female	34,236.7(53.7)	10,625.2(53.6)	10,373.5(53.6)	11,515.7(53.2)	33,244.0(54.1)	
Ethnic						0.002
Han	63,192.1(99.1)	19,645.9(99.1)	19,179.4(99.1)	21,459.0(99.1)	60,922.6(99.1)	
Minorities	586.9(0.9)	177.5(0.9)	174.2(0.9)	199.1(0.9)	571.3(0.9)	
Marital status						0.048
Never married	16,676.1(26.1)	5,135.7(25.9)	5,110.7(26.4)	5,663.6(26.1)	17,535.4(28.5)	
Married	40,925.2(64.2)	12,840.2(64.8)	12,391.1(64.0)	13,921.0(64.3)	36,826.1(59.9)	
Widowed	2,680.4(4.2)	847.2(4.3)	835.9(4.3)	965.2(4.5)	3,338.0(5.4)	
Divorced	3,497.4(5.5)	1,000.2(5.0)	1,015.8(5.2)	1,108.4(5.1)	3,794.4(6.2)	
Urbanicity						0.007
Rural	51,691.5(81.0)	16,072.4(81.1)	15,698.2(81.1)	17,521.4(80.9)	49,520.7(80.5)	
Urban	12,087.5(19.0)	3,751.0(18.9)	3,655.3(18.9)	4,136.7(19.1)	11,973.2(19.5)	
Education level						0.014
Primary school or lower	4,2440.4(66.5)	13,189.8(66.5)	12,893.5(66.6)	14,404.3(66.5)	40,857.7(66.4)	
Middle and high school	20,095.8(31.5)	6,212.5(31.3)	6,029.4(31.2)	6,785.2(31.3)	19,174.3(31.2)	
College/university or	1,242.8(1.9)	421.1(2.1)	430.6(2.2)	468.7(2.2)	1,461.9(2.4)	

higher						
Economic situation						0.004
Poverty †	41,201.7(64.6)	12,806.6(64.6)	12,524.5(64.7)	13,960.7(64.5)	39,511.5(64.3)	
Non-poverty	22,577.3(35.4)	7,016.8(35.4)	6,829.1(35.3)	7,697.4(35.5)	21,982.4(35.7)	
**Clinical characteristics**						
Family history of psychiatric illness						0.005
Yes	2,985.7(4.7)	936.6(4.7)	885.3(4.6)	1,004.5(4.6)	2,941.7(4.8)	
No	60,793.3(95.3)	18,886.7(95.3)	18,468.2(95.4)	20,653.6(95.4)	58,552.1(95.2)	
Duration of illness						
mean (SD), years	12.45(11.14)	12.69(11.17)	12.71(11.17)	12.59(11.24)	12.62(11.04)	0.011
<10	32,535.2(51.0)	9,889.4(49.9)	9,700.0(50.1)	11,075.1(51.1)	31,228.9(50.8)	0.021
10~19	16,467.2(25.8)	5,234.7(26.4)	5,077.4(26.2)	5,560.7(25.7)	16,140.2(26.2)	
20~29	9,223.7(14.5)	2,955.6(14.9)	2,853.0(14.7)	3,028.2(14.0)	8,815.2(14.3)	
≥30	5,553.0(8.7)	1,743.6(8.8)	1,723.1(8.9)	1,994.2(9.2)	5,309.5(8.6)	
**History of self-inflicted violence behaviors**						
History of suicide attempt at baseline						0.008
Yes	250.4(0.4)	100.6(0.5)	96.7(0.5)	102.2(0.5)	307.1(0.5)	
No	59,056.9(92.6)	18,336.6(92.5)	17,913.7(92.6)	20,049.0(92.6)	56,937.6(92.6)	
Unknown	4,471.8(7.0)	1,386.1(7.0)	1,343.3(6.9)	1,506.9(7.0)	4,249.1(6.9)	
History of NSSI at baseline						0.007

Yes	248.1(0.4)	88.6(0.4)	84.0(0.4)	92.3(0.4)	297.6(0.5)
No	58,842.1(92.3)	18,274.9(92.2)	17,856.9(92.3)	19,981.9(92.3)	56,743.8(92.3)
Unknown	4,688.9(7.4)	1,459.9(7.4)	1,412.7(7.3)	1,584.0(7.3)	4,452.5(7.2)

Table S9. Association between 2 levels of medication adherence and self-inflicted violence behaviors (follow-up of ≥1 year for hypothesis 1)

Self-inflicted violence behaviors	Statistics	Medication adherence	Medication non-adherence	*P* value
**Suicide**				
	Hazard Ratio (95% CI) †	ref	0.639(0.525,0.776)	<0.001
	Restricted mean survival time at 2738 days (95% CI)			
	Difference—days	ref	2.652(1.263,4.042)	<0.001
	Ratio	ref	1.001(1.000,1.001)	<0.001
**Suicide attempt**				
	Hazard Ratio (95% CI) †	ref	1.115(0.924,1.346)	0.260
	Restricted mean survival time at 2570 days (95% CI)			
	Difference—days	ref	-1.037(-2.316,0.242)	0.112
	Ratio	ref	1.000(0.999,1.000)	0.112
**NSSI**				
	Hazard Ratio (95% CI) †	ref	1.143(0.963,1.358)	0.130
	Restricted mean survival time at 2570 days (95% CI)			
	Difference—days	ref	-1.941(-3.359, -0.522)	0.007
	Ratio	ref	0.999(0.999,1.000)	0.007

† The model included region as a random effect. The variance (standard deviation) of the random effect of region was 0.05 (0.21) for suicide, 0.17 (0.42) for suicide attempt, and 0.65 (0.80) for NSSI.

Table S10. Association between 5 levels of medication adherence and self-inflicted violence behaviors (follow-up of ≥1 year for hypothesis 2)

Self-inflicted violence behaviors	Statistics	P5	P4	P3	P2	P1
**Suicide**						
	Hazard Ratio (95% CI) †	ref	0.616(0.439,0.866)**	0.598(0.424,0.843)**	0.715(0.528,0.968)*	0.645(0.512,0.813)**
	Restricted mean survival time at 2534 days (95% CI)					
	Difference—days	ref	2.456(0.827,4.084)**	2.599(0.969,4.229)**	1.981(0.336,3.626)*	2.245(0.881,3.608)**
	Ratio	ref	1.001(1.000,1.002)**	1.001(1.000,1.002)**	1.001(1.000,1.001)*	1.001(1.000,1.001)**
**Suicide attempt**						
	Hazard Ratio (95% CI) †	ref	0.761(0.547,1.059)	0.840(0.612,1.153)	1.000(0.754,1.327)	1.430(1.168,1.750)***
	Restricted mean survival time at 2345 days (95% CI)					
	Difference—days	ref	1.561(0.158,2.964)*	0.705(-0.823,2.233)	-0.405(-2.005,1.196)	-2.858(-4.286, -1.430)***
	Ratio	ref	1.001(1.000,1.001)	1.000(1.000,1.001)	1.000(0.999,1.001)	0.999(0.998,0.999)
**NSSI**						
	Hazard Ratio (95% CI) †	ref	0.808(0.601,1.085)	0.703(0.518,0.954)*	1.042(0.810,1.339)	1.472(1.225,1.769)***
	Restricted mean survival time at 2345 days (95% CI)					
	Difference—days	ref	1.062(-0.575,2.700)	1.421(-0.188,3.030)	-1.125(-2.943,0.693)	-4.302(-5.913, -2.692)***
	Ratio	ref	1.000(1.000,1.001)	1.001(1.000,1.001)	1.000(0.999,1.000)	0.998(0.998,0.999)***

† The model included region as a random effect. The variance (standard deviation) of the random effect of region was 0.05(0.22) for suicide, 0.16(0.40) for suicide attempt, and 0.69(0.83) for NSSI.

P* value <0.05, ** *P* value <0.01, * *P* value <0.001.

Table S11. Association between 2 levels of medication adherence and self-inflicted violence behaviors (the number of records of medication adherence ≥ 5 for hypothesis 1)

Self-inflicted violence behaviors	Statistics	Medication adherence	Medication non-adherence	*P* value
**Suicide**				
	Hazard Ratio (95% CI) †	ref	0.601(0.494,0.730)	<0.001
	Restricted mean survival time at 2738 days (95% CI)			
	Difference—days	ref	3.093(1.682,4.504)	<0.001
	Ratio	ref	1.001(1.001,1.002)	<0.001
**Suicide attempt**				
	Hazard Ratio (95% CI) †	ref	1.219(0.997,1.491)	0.054
	Restricted mean survival time at 2509 days (95% CI)			
	Difference—days	ref	-1.309(-2.464,-0.154)	0.026
	Ratio	ref	0.999(0.999,1.000)	0.026
**NSSI**				
	Hazard Ratio (95% CI) †	ref	1.337(1.107,1.615)	0.003
	Restricted mean survival time at 2398 days (95% CI)			
	Difference—days	ref	-2.463(-3.618, -1.308)	<0.001
	Ratio	ref	0.999(0.998,0.999)	<0.001

† The model included region as a random effect. The variance (standard deviation) of the random effect of region was 0.03 (0.18) for suicide, 0.15 (0.38) for suicide attempt, and 0.57 (0.75) for NSSI.

Table S12. Association between 5 levels of medication adherence and self-inflicted violence behaviors (the number of records of medication adherence ≥ 5 for hypothesis 2)

Self-inflicted violence behaviors	Statistics	P5	P4	P3	P2	P1
**Suicide**						
	Hazard Ratio (95% CI) †	ref	0.605(0.432,0.849)**	0.554(0.391,0.786)***	0.697(0.515,0.943)*	0.584(0.462,0.739)***
	Restricted mean survival time at 2534 days (95% CI)					
	Difference—days	ref	2.666(1.011,4.321)**	3.082(1.464,4.700)***	2.207(0.534,3.880)*	2.819(1.451,4.187)***
	Ratio	ref	1.001(1.000,1.002)**	1.001(1.001,1.002)***	1.001(1.000,1.002)*	1.001(1.001,1.002)***
**Suicide attempt**						
	Hazard Ratio (95% CI) †	ref	0.933(0.669,1.300)	0.988(0.715,1.365)	1.107(0.823,1.489)	1.487(1.197,1.849)***
	Restricted mean survival time at 2345 days (95% CI)					
	Difference—days	ref	0.682(-0.720,2.084)	0.037(-1.463,1.536)	-0.694(-2.222,0.833)	-2.632(-3.981, -1.282)***
	Ratio	ref	1.000(1.000,1.001)	1.000(0.999,1.001)	1.000(0.999,1.000)	0.999(0.998,0.999)***
**NSSI**						
	Hazard Ratio (95% CI) †	ref	1.129(0.841,1.515)	0.964(0.710,1.310)	1.240(0.949,1.620)	1.602(1.307,1.963)***
	Restricted mean survival time at 2345 days (95% CI)					
	Difference—days	ref	-0.781(-2.459,0.897)	-0.219(-1.848,1.410)	-1.730(-3.447,-0.013)*	-4.090(-5.583, -2.597)***
	Ratio	ref	1.000(0.999,1.000)	1.000(0.999,1.001)	0.999(0.999,1.000)*	0.998(0.998,0.999)***

† The model included region as a random effect. The variance (standard deviation) of the random effect of region was 0.03(0.18) for suicide, 0.14(0.37) for suicide attempt, and 0.62(0.79) for NSSI.

P* value <0.05, ** *P* value <0.01, * *P* value <0.001.

Table S13. Association between 2 levels of medication adherence and self-inflicted violence behaviors (Re-define the PRM for hypothesis 1)

Self-inflicted violence behaviors	Statistics	Medication adherence	Medication non-adherence	*P* value
**Suicide**				
	Hazard Ratio (95% CI) †	ref	0.527(0.447, 0.621)	<0.001
	Restricted mean survival time at 2738 days (95% CI)			
	Difference—days	ref	5.444(3.759, 7.128)	0.001
	Ratio	ref	1.002(1.001, 1.003)	0.001
**Suicide attempt**				
	Hazard Ratio (95% CI) †	ref	1.012(0.890, 1.151)	0.860
	Restricted mean survival time at 2570 days (95% CI)			
	Difference—days	ref	-0.820(-2.727, 1.086)	0.399
	Ratio	ref	1.000 (0.999, 1.000)	0.399
**NSSI**				
	Hazard Ratio (95% CI) †	ref	1.239(1.097, 1.400)	<0.001
	Restricted mean survival time at 2570 days (95% CI)			
	Difference—days	ref	-4.853(-6.887, -2.818)	<.001
	Ratio	ref	0.998(0.997, 0.999)	<.001

† The model included region as a random effect. The variance (standard deviation) of the random effect of region was 0.04 (0.20) for suicide, 0.12 (0.34) for suicide attempt, and 0.30 (0.55) for NSSI.

Table S14. Association between 5 levels of medication adherence and self-inflicted violence behaviors (Re-define the PRM for hypothesis 2)

Self-inflicted violence behaviors	Statistics	P5	P4	P3	P2	P1
**Suicide**						
	Hazard Ratio (95% CI) †	ref	0.489(0.360,0.663)***	0.441(0.321,0.607)***	0.560(0.426,0.735)***	0.597(0.493,0.723)***
	Restricted mean survival time at 2534 days (95% CI)					
	Difference—days	ref	5.346(3.418,7.274)***	5.917(4.034,7.801)***	4.852(2.905,6.799)***	4.026(2.309,5.744)***
	Ratio	ref	1.002(1.001,1.003)***	1.002(1.002,1.003)***	1.002(1.001,1.003)***	1.002(1.001,1.002)***
**Suicide attempt**						
	Hazard Ratio (95% CI) †	ref	0.498(0.379,0.653)***	0.574(0.445,0.741)***	0.570(0.448,0.727)***	1.642(1.436,1.877)***
	Restricted mean survival time at 2345 days (95% CI)					
	Difference—days	ref	6.722(4.775,8.668)***	5.273(3.156,7.391)***	5.456(3.433,7.480)***	-9.457 (-11.857, -7.056)***
	Ratio	ref	1.003(1.002,1.004)***	1.002(1.001,1.003)***	1.002(1.002,1.003)***	0.996(0.995,0.997)***
**NSSI**						
	Hazard Ratio (95% CI) †	ref	0.698(0.555,0.879)**	0.577(0.451,0.738)***	0.713(0.576,0.883)**	1.910(1.682,2.169)***
	Restricted mean survival time at 2345 days (95% CI)					
	Difference—days	ref	3.889(1.603,6.175)**	5.093(2.887,7.300)***	3.565(1.312,5.818)**	-14.268(-16.872, -11.664)***
	Ratio	ref	1.002(1.001,1.003)**	1.002(1.001,1.003)***	1.002(1.001,1.002)**	0.994(0.993,0.995)***

† The model included region as a random effect. The variance (standard deviation) of the random effect of region was 0.04(0.2 0) for suicide, 0.13(0.35) for suicide attempt, and 0.31(0.55) for NSSI.

P* value <0.05, ** *P* value <0.01, * *P* value <0.001.

Table S15. E-value for quantifying unmeasured confounders

	E-value
**Suicide**	
P4	3.51
P3	3.94
P2	2.95
P1	2.76
Medication non-adherence (Binary)	3.20
**Suicide attempt**	
P4	3.46
P3	2.79
P2	2.90
P1	2.61
Medication non-adherence (Binary)	-
**NSSI**	
P4	2.16
P3	2.87
P2	2.02
P1	3.15
Medication non-adherence (Binary)	1.76

The HRs for E-values calculation were obtained from inverse probability-weighted Cox proportional hazards model with mixed-effects

Table S16. Sex-stratified association between 2 levels of medication adherence and self-inflicted violence behaviors during follow-up period (for hypothesis 1)

Self-inflicted violence behaviors	Statistics	Male			Female		
		Medication adherence	Medication non-adherence	*P* value	Medication adherence	Medication non-adherence	*P* value
**Suicide**							
	Hazard Ratio (95% CI) †	ref	0.607(0.477,0.771)	<0.001	ref	0.468(0.373,0.586)	<0.001
	Restricted mean survival time at 3000‡/2210§ days (95% CI)						
	Difference—days	ref	5.178(2.138,8.218)	0.001	ref	4.724(3.067,6.382)	<.001
	Ratio	ref	1.002(1.001,1.003)	0.001	ref	1.002(1.001,1.003)	<.001
**Suicide attempt**							
	Hazard Ratio (95% CI) †	ref	0.979(0.813,1.178)	0.820	ref	1.035 (0.866,1.237)	0.710
	Restricted mean survival time at 2509‡/2570§ days (95% CI)						
	Difference—days	ref	-0.220(-2.967,2.526)	0.875	ref	-1.169(-3.747,1.409)	0.374
	Ratio	ref	1.000(0.999,1.001)	0.875	ref	1.000(0.999,1.001)	0.374
**NSSI**							
	Hazard Ratio (95% CI) †	ref	1.259(1.059,1.496)	0.009	ref	1.210(1.019,1.437)	0.029
	Restricted mean survival time at 2398‡/2570§ days (95% CI)						
	Difference—days	ref	-4.965(-7.751, -2.179)	<0.001	ref	-4.327(-7.04, -1.613)	0.002
	Ratio	ref	0.998(0.997,0.999)	<0.001	ref	0.998(0.997,0.999)	0.002

† The model included region as a random effect. In male patients, the variance (standard deviation) of the random effect of region was 0.05(0.23) for suicide, 0.07 (0.26) for suicide attempt, and 0.21(0.46) for NSSI. In female patients, the variance (standard deviation) of the random effect of region was 0.05(0.21) for suicide, 0.19(0.43) for suicide attempt, and 0.44(0.66) for NSSI.

‡ Truncation time for male patients.

§ Truncation time for female patients.

Table S17. Association between 5 levels of medication adherence and self-inflicted violence behaviors among male patients (for hypothesis 2)

Self-inflicted violence behaviors	Statistics	P5	P4	P3	P2	P1
**Suicide**						
	Hazard Ratio (95% CI) †	ref	0.444(0.273,0.721)**	0.529(0.336,0.835)**	0.758(0.522,1.100)	0.662(0.502,0.874)**
	Restricted mean survival time at 1904 days (95% CI)					
	Difference—days	ref	3.181(1.475,4.888)***	3.008(1.213,4.804)**	1.792(-0.162,3.746)	2.013(0.446,3.581)*
	Ratio	ref	1.002(1.001,1.003)***	1.002(1.000,1.003)**	1.001(1.000,1.002)	1.001(1.000,1.002)*
**Suicide attempt**						
	Hazard Ratio (95% CI) †	ref	0.486(0.325,0.727)***	0.682(0.479,0.969)*	0.611(0.431,0.867)**	1.431(1.177,1.741)***
	Restricted mean survival time at 2222 days (95% CI)					
	Difference—days	ref	6.855(4.201,9.508)***	3.690(0.526,6.854)*	4.992(2.140,7.844)**	-6.583(-9.753, -3.414)***
	Ratio	ref	1.003(1.002,1.004)***	1.002(1.000,1.003)*	1.002(1.001,1.004)**	0.997(0.996,0.998)***
**NSSI**						
	Hazard Ratio (95% CI) †	ref	0.729(0.527,1.011)	0.669(0.477,0.937)*	0.956 (0.724,1.264)	1.755(1.461,2.106)***
	Restricted mean survival time at 2289 days (95% CI)					
	Difference—days	ref	3.727(0.324,7.130)*	3.735(0.256,7.213)*	0.393(-3.315,4.102)	-12.309(-15.956, -8.662)***
	Ratio	ref	1.002(1.000,1.003)*	1.002(1.000,1.003)*	1.000(0.999,1.002)	0.995(0.993,0.996)***

† The model included region as a random effect. The variance (standard deviation) of the random effect of region was 0.06(0.25) for suicide, 0.06(0.24) for suicide attempt, and 0.19(0.44) for NSSI.

P* value <0.05, ** *P* value <0.01, * *P* value <0.001.

Table S18. Association between 5 levels of medication adherence and self-inflicted violence behaviors among female patients (for hypothesis 2)

Self-inflicted violence behaviors	Statistics	P5	P4	P3	P2	P1
**Suicide**						
	Hazard Ratio (95% CI) †	ref	0.529(0.358,0.782) **	0.375(0.239,0.588) ***	0.404(0.268,0.609) ***	0.548(0.421,0.714) ***
	Restricted mean survival time at 2210 days (95% CI)					
	Difference—days	ref	4.538(2.402,6.673)***	5.549(3.546,7.552)***	5.485(3.507,7.463)***	3.745(1.816,5.674)***
	Ratio	ref	1.002(1.001,1.003)***	1.003(1.002,1.003)***	1.002(1.002,1.003)***	1.002(1.001,1.003)***
**Suicide attempt**						
	Hazard Ratio (95% CI) †	ref	0.506(0.350,0.732)***	0.503(0.349,0.724)***	0.535(0.382,0.749)***	1.791(1.491,2.151)***
	Restricted mean survival time at 2316 days (95% CI)					
	Difference—days	ref	6.111(3.501,8.721)***	5.914(3.232,8.595)***	5.516(2.874,8.159)***	-10.962(-14.258, -7.666)***
	Ratio	ref	1.003(1.002,1.004)***	1.003(1.001,1.004)***	1.002(1.001,1.004)***	0.995(0.994,0.997)***
**NSSI**						
	Hazard Ratio (95% CI) †	ref	0.691(0.502,0.951)*	0.493(0.344,0.706)***	0.546(0.394,0.756)***	1.996(1.671,2.384)***
	Restricted mean survival time at 2316 days (95% CI)					
	Difference—days	ref	3.492(0.495,6.490)*	5.963(3.256,8.670)***	5.243(2.510,7.976)***	-14.762(-18.268, -11.256)***
	Ratio	ref	1.002(1.000,1.003)*	1.003(1.001,1.004)***	1.002(1.001,1.004)***	0.994(0.992,0.995)***

† The model included region as a random effect. The variance (standard deviation) of the random effect of region was 0.03(0.17) for suicide, 0.21(0.46) for suicide attempt, and 0.48(0.69) for NSSI.

P* value <0.05, ** *P* value <0.01, * *P* value <0.001.

Table S19. Urbanity-stratified association between 2 levels of medication adherence and self-inflicted violence behaviors during follow-up period **(for hypothesis 1)**

Self-inflicted violence behaviors	Statistics	Rural			Urban		
		Medication adherence	Medication non-adherence	*P* value	Medication adherence	Medication non-adherence	*P* value
**Suicide**							
	Hazard Ratio (95% CI) †	ref	0.473(0.392,0.572)	<0.001	ref	0.839(0.577,1.219)	0.360
	Restricted mean survival time at 2738‡/2265§ days (95% CI)						
	Difference—days	ref	6.356(4.380,8.332)	<0.001	ref	0.619(-1.847,3.084)	0.623
	Ratio	ref	1.002(1.002,1.003)	<0.001	ref	1.000(0.999,1.001)	0.623
**Suicide attempt**							
	Hazard Ratio (95% CI) †	ref	0.972(0.835,1.132)	0.710	ref	1.142(0.863,1.511)	0.350
	Restricted mean survival time at 2570‡/2359§ days (95% CI)						
	Difference—days	ref	-0.178(-2.344,1.988)	0.872	ref	-2.541(-6.297,1.214)	0.185
	Ratio	ref	1.000(0.999,1.001)	0.872	ref	0.999(0.997,1.001)	0.185
**NSSI**							
	Hazard Ratio (95% CI) †	ref	1.226(1.063,1.413)	0.005	ref	1.309(0.975,1.759)	0.073
	Restricted mean survival time at 2570‡/2199§ days (95% CI)						
	Difference—days	ref	-4.902(-7.243, -2.560)	<0.001	ref	-3.729(-7.03, -0.429)	0.027
	Ratio	ref	0.998(0.997,0.999)	<0.001	ref	0.998(0.997,1.000)	0.027

† The model included region as a random effect. In rural patients, the variance (standard deviation) of the random effect of region was 0.02 (0.14) for suicide, 0.10 (0.32) for suicide attempt, and 0.30 (0.54) for NSSI. In urban patients, the variance (standard deviation) of the random effect of region was 0.11(0.33) for suicide, 0.38(0.62) for suicide attempt, and 0.27(0.52) for NSSI.

‡ Truncation time for rural patients.

§ Truncation time for urban patients.

Table S20. Association between 5 levels of medication adherence and self-inflicted violence behaviors among rural patients (for hypothesis 2)

Self-inflicted violence behaviors	Statistics	P5	P4	P3	P2	P1
**Suicide**						
	Hazard Ratio (95% CI) †	ref	0.480(0.343,0.671)***	0.429(0.304,0.605)***	0.454(0.331,0.623)***	0.518(0.416,0.644)***
	Restricted mean survival time at 2534 days (95% CI)					
	Difference—days	ref	5.638(3.407,7.870)***	6.405(4.285,8.524)***	6.158(4.069,8.246)***	5.121(3.241,7.001)***
	Ratio	ref	1.002(1.001,1.003)***	1.003(1.002,1.003)***	1.002(1.002,1.003)***	1.002(1.001,1.003)***
**Suicide attempt**						
	Hazard Ratio (95% CI) †	ref	0.428(0.310,0.590)***	0.533(0.399,0.710)***	0.602(0.465,0.781)***	1.487(1.270,1.741)***
	Restricted mean survival time at 2345 days (95% CI)					
	Difference—days	ref	7.584(5.42,9.746)***	5.854(3.521,8.188)***	5.287(3.033,7.540)***	-7.416(-9.815, -5.016)***
	Ratio	ref	1.003(1.002,1.004)***	1.003(1.002,1.004)***	1.002(1.001,1.003)****	0.997(0.996,0.998)***
**NSSI**						
	Hazard Ratio (95% CI) †	ref	0.629(0.484,0.818)***	0.557(0.39*,0.6//)***	0.796(0.638,0.993)***	1.837(1.587,2.128)***
	Restricted mean survival time at 2345 days (95% CI)					
	Difference—days	ref	4.878(2.301,7.455)***	6.076(3.637,8.515)***	2.632(0.049,5.215)*	-13.814(-16.480, -11.147)***
	Ratio	ref	1.002(1.001,1.003)***	1.003(1.002,1.004)***	1.001(1.000,1.002)*	0.994(0.993,0.995)***

† The model included region as a random effect. The variance (standard deviation) of the random effect of region was 0.02(0.15) for suicide, 0.09(0.30) for suicide attempt, and 0.29(0.53) for NSSI.

P* value <0.05, ** *P* value <0.01, * *P* value <0.001.

Table S21. Association between 5 levels of medication adherence and self-inflicted violence behaviors among urban patients (for hypothesis 2)

Self-inflicted violence behaviors	Statistics	P5	P4	P3	P2	P1
**Suicide**						
	Hazard Ratio (95% CI) †	ref	0.525(0.243,1.136)	0.524(0.217,1.266)	1.308(0.722,2.368)	1.058(0.647,1.731)
	Restricted mean survival time at 1259 days (95% CI)					
	Difference—days	ref	1.468(0.451,2.486)**	0.855(-0.655,2.364)	0.284(-1.403,1.971)	-0.779(-2.740,1.182)
	Ratio	ref	1.001(1.000,1.002)**	1.001(0.999,1.002)	1.000(0.999,1.002)	0.999(0.998,1.001)
**Suicide attempt**						
	Hazard Ratio (95% CI) †	ref	0.822(0.485,1.391)	0.840(0.468,1.508)	0.518(0.247,1.085)	2.099(1.538,2.866)***
	Restricted mean survival time at 1521 days (95% CI)					
	Difference—days	ref	2.673(0.312,5.214)*	2.210(-0.596,5.015)	3.243(0.593,5.893)*	-9.088(-13.891, -4.284)***
	Ratio	ref	1.002(1.000,1.003)*	1.001(1.000,1.003)	1.002(1.000,1.004)*	0.994(0.991,0.997)***
**NSSI**						
	Hazard Ratio (95% CI) †	ref	1.258(0.777,2.037)	0.935 (0.504,1.734)	0.513(0.227,1.159)	2.138(1.523,3.002)***
	Restricted mean survival time at 1153 days (95% CI)					
	Difference—days	ref	-0.266(-2.363,1.831)	0.536(-1.546,2.617)	1.237(-0.734,3.208)	-5.251(-8.226, -2.277)**
	Ratio	ref	1.000(0.998,1.002)	1.000(0.999,1.002)	1.001(0.999,1.003)	0.995(0.993,0.998)**

† The model included region as a random effect. The variance (standard deviation) of the random effect of region was 0.10 (0.31) for suicide, 0.39(0.63) for suicide attempt, and 0.29(0.54) for NSSI.

P* value <0.05, ** *P* value <0.01, * *P* value <0.001.

Table S22. Age-stratified association between 2 levels of medication adherence and self-inflicted violence behaviors during follow-up period (for hypothesis 1)

Self-inflicted violence behaviors	Statistics	Medication adherence	Medication non-adherence			
			10 to 24	25 to 44	45 to 59	≥60
**Suicide**						
	Hazard Ratio (95% CI) †	ref	0.514(0.301,0.879)*	0.730(0.569,0.936)*	0.433(0.323,0.579)***	0.332(0.217,0.512)***
	Restricted mean survival time at 2265‡/2357§/2321¶/2090✧ days (95% CI)					
	Difference—days	ref	6.364(1.049,11.678)*	2.165(0.283,4.046)*	5.538(3.102,7.974)***	5.066(2.477,7.655)***
	Ratio	ref	1.003(1.000,1.005)*	1.001(1.000,1.002)*	1.002(1.001,1.003)***	1.002(1.001,1.004)***
**Suicide attempt**						
	Hazard Ratio (95% CI) †	ref	1.068(0.733,1.155)	0.981(0.819,1.175)	1.375(1.046,1.807)*	0.729(0.525,1.013)
	Restricted mean survival time at 2139‡/2509§/2168¶/2570✧ days (95% CI)					
	Difference—days	ref	-2.596(-9.373,4.182)	-0.191(-3.230,2.848)	-2.690(-5.055,-0.325)*	3.099(-1.206,7.404)
	Ratio	ref	0.999(0.996,1.002)	1.000(0.999,1.001)	0.999(0.998,1.000)*	1.001(1.000,1.003)
**NSSI**						
	Hazard Ratio (95% CI) †	ref	1.021(0.716,1.455)	1.299(1.095,1.540)**	1.375(1.074,1.760)*	1.054(0.760,1.462)
	Restricted mean survival time at 2139‡/2398§ /1994¶/2570✧ days (95% CI)					
	Difference—days	ref	-2.270(-9.454, 4.914)	-6.189(-9.246,-3.132)***	-3.746(-6.059,-1.433)**	-1.680(-5.988,2.627)
	Ratio	ref	0.999(0.996,1.002)	0.997(0.996,0.999)***	0.998(0.997,0.999)**	0.999(0.998,1.001)

† The model included region as a random effect. In patients aged 10 to 24, the variance (standard deviation) of the random effect of region was 0.02 (0.47) for suicide, 0.02 (0.50) for suicide attempt, and 0.38 (0.61) for NSSI. In patients aged 25 to 44, the variance (standard deviation) of the random effect of region was 0.06(0.25) for suicide, 0.11(0.34) for suicide attempt, and 0.34(0.58) for NSSI. In patients aged 45 to 59, the variance (standard deviation) of the random effect of region was 0.09(0.30) for suicide,

0.08(0.29) for suicide attempt, and 0.24(0.49) for NSSI. In patients aged ≥60, the variance (standard deviation) of the random effect of region was 0.08(0.28) for suicide, 0.23(0.48) for suicide attempt, and 0.47(0.68) for NSSI.

‡ Truncation time for patients aged 10 to 24.

§ Truncation time for patients aged 25 to 44.

¶ Truncation time for patients aged 45 to 59.

✧ Truncation time for patients aged ≥60

P* value <0.05, ** *P* value <0.01, * *P* value <0.001.

Table S23. Association between 5 levels of medication adherence and self-inflicted violence behaviors among patients aged 10 to 24 (for hypothesis 2)

Self-inflicted violence behaviors	Statistics	P5	P4	P3	P2	P1
**Suicide**						
	Hazard Ratio (95% CI) †	ref	0.547(0.223,1.340)	0.509(0.191,1.356)	0.326(0.102,1.043)	0.561(0.271,1.160)
	Restricted mean survival time at 1678 days (95% CI)					
	Difference—days	ref	4.692(0.813,8.571)*	3.700(-0.942,8.343)	4.787(0.613,8.961)*	2.570(-1.873,7.013)
	Ratio	ref	1.003(1.000,1.005)*	1.002(0.999,1.005)	1.003(1.000,1.005)*	1.002(0.999,1.004)
**Suicide attempt**						
	Hazard Ratio (95% CI) †	ref	0.495(0.228,1.075)	1.188(0.670,2.106)	0.320(0.122,0.837)*	1.872(1.239,2.828)**
	Restricted mean survival time at 897 days (95% CI)					
	Difference—days	ref	1.968(-0.006,3.942)	0.721-1.929,3.371)	2.390(0.609,4.171)**	-6.936(-10.999, -2.873)**
	Ratio	ref	1.002(1.000,1.004)	1.001(0.998,1.004)	1.003(1.001,1.005)**	0.992(0.988,0.997)**
**NSSI**						
	Hazard Ratio (95% CI) †	ref	0.629(0.326,1.211)	0.576(0.283,1.172)	0.829(0.458,1.501)	1.649(1105,2.460)*
	Restricted mean survival time at 1786 days (95% CI)					
	Difference—days	ref	5.216(-1.518,11.949)	5.073(-2.206,12.353)	-0.423(-9.279,8.433)	-11.315(-20.699, -1.930)*
	Ratio	ref	1.003(0.999,1.007)	1.003(0.999,1.007)	1.000(0.995,1.005)	0.994(0.988,0.999)*

† The model included region as a random effect. The variance (standard deviation) of the random effect of region was 0.20 (0.45) for suicide, 0.29(0.54) for suicide attempt, and 0.38(0.62) for NSSI.

P* value <0.05, ** *P* value <0.01, * *P* value <0.001.

Table S24. Association between 5 levels of medication adherence and self-inflicted violence behaviors among patients aged 25 to 44 (for hypothesis 2)

Self-inflicted violence behaviors	Statistics	P5	P4	P3	P2	P1
**Suicide**						
	Hazard Ratio (95% CI) †	ref	0.562(0.354,0.892)*	0.594(0.374,0.943)*	0.763(0.509,1.146)	0.889(0.661,1.192)
	Restricted mean survival time at 2320 days (95% CI)					
	Difference—days	ref	3.328(0.919,5.737)**	3.359(0.907,5.812)**	2.370(-0.251,4.991)	0.623(-1.795,3.041)
	Ratio	ref	1.001(1.000,1.002)**	1.001(1.000,1.003)**	1.001(1.000,1.002)	1.000(0.999,1.001)
**Suicide attempt**						
	Hazard Ratio (95% CI) †	ref	0.470(0.318,0.693)***	0.523(0.358,0.765)***	0.588(0.414,0.836)**	1.686(1.392,2.043)***
	Restricted mean survival time at 2345 days (95% CI)					
	Difference—days	ref	8.269(5.089,11.449)***	7.507(4.073,10.941)***	6.988(3.610,10.366)***	-11.833(-16.232, -7.435)***
	Ratio	ref	1.004(1.002,1.005)***	1.003(1.002,1.005)***	1.003(1.002,1.004)***	0.995(0.993,0.997)***
**NSSI**						
	Hazard Ratio (95% CI) †	ref	0.619(0.0.440,0.870)**	0.538 (0.374,0.776)***	0.876(0.655,1.172)	2.056(1.715,2.464)***
	Restricted mean survival time at 1884 days (95% CI)					
	Difference—days	ref	4.220(1.637,6.803)**	4.673(2.029,7.318)**	2.515(-0.323,5.352)	-15.143(-18.806, -11.481)***
	Ratio	ref	1.002(1.001,1.004)**	1.002(1.001,1.004)**	1.001(1.000,1.003)	0.992(0.990,0.994)**

† The model included region as a random effect. The variance (standard deviation) of the random effect of region was 0.06(0.25) for suicide, 0.12 (0.34) for suicide attempt, and 0.33(0.57) for NSSI.

*P value <0.05, ** P value <0.01, *** P value <0.001.

Table S25. Association between 5 levels of medication adherence and self-inflicted violence behaviors among patients aged 45 to 59 (for hypothesis 2)

Self-inflicted violence behaviors	Statistics	P5	P4	P3	P2	P1
**Suicide**						
	Hazard Ratio (95% CI) †	ref	0.340(0.186,0.623)***	0.376(0.212,0.667)***	0.415(0.249,0.693)***	0.522(0.374,0.728)***
	Restricted mean survival time at 2237 days (95% CI)					
	Difference—days	ref	6.035(3.292,8.779)***	5.545(2.652,8.438)***	5.487(2.697,8.276)***	4.247(1.711,6.782)***
	Ratio	ref	1.003(1.001,1.004)***	1.002(1.001,1.004)***	1.002(1.001,1.004)***	1.002(1.001,1.003)***
**Suicide attempt**						
	Hazard Ratio (95% CI) †	ref	0.595(0.342,1.035)	0.701(0.419,1.170)	0.677(0.415,1.103)	2.188(1.652,2.898)***
	Restricted mean survival time at 1924 days (95% CI)					
	Difference—days	ref	3.705 (1.512,5.898)**	2.356(-0.167,4.878)	2.344(-0.041,4.729)	-7.586(-10.398, -4.774)***
	Ratio	ref	1.002(1.001,1.003)**	1.001(1.000,1.003)	1.001(1.000,1.002)	0.996(0.995,0.998)***
**NSSI**						
	Hazard Ratio (95% CI) †	ref	0.961(0.634,1.457)	0.897(0.588,1.368)	0.670(0.431,1.042)	2.024(1.566,2.612)***
	Restricted mean survival time at 1984 days (95% CI)					
	Difference—days	ref	0.488(-2.738,3.715)	1.129(-1.983,4.241)	2.354(-0.453,5.160)	-9.542(-12.727, -6.356)***
	Ratio	ref	1.000(0.999,1.002)	1.001(0.999,1.002)	1.001(1.000,1.003)	0.995(0.994,0.997)***

† The model included region as a random effect. The variance (standard deviation) of the random effect of region was 0.11 (0.33) for suicide, 0.10(0.32) for suicide attempt, and 0.25(0.50) for NSSI.

P* value <0.05, ** *P* value <0.01, * *P* value <0.001.

Table S26. Association between 5 levels of medication adherence and self-inflicted violence behaviors among patients aged ≥60 (for hypothesis 2)

Self-inflicted violence behaviors	Statistics	P5	P4	P3	P2	P1
**Suicide**						
	Hazard Ratio (95% CI) †	ref	0.578(0.288,1.160)	0.259(0.102,0.658)**	0.591(0.322,1.084)	0.212(0.120,0.374)***
	Restricted mean survival time at 1518 days (95% CI)					
	Difference—days	ref	2.312(0.441,4.182)*	3.484(1.880,5.087)***	2.257(0.376,4.138)*	3.271(1.731,4.811)***
	Ratio	ref	1.002(1.000,1.003)*	1.002(1.001,1.003)***	1.001(1.000,1.003)*	1.002(1.001,1.003)***
**Suicide attempt**						
	Hazard Ratio (95% CI) †	ref	0.464(0.235,0.915)*	0.369(0.180,0.756)**	0.614(0.359,1.050)	0.905(0.640,1.278)
	Restricted mean survival time at 1120 days (95% CI)					
	Difference—days	ref	1.960(0.534,3.386)**	1.814(0.378,3.251)*	1.566(0.164,2.968)*	-0.873(-2.306, 0.561)
	Ratio	ref	1.002(1.000,1.003)**	1.002(1.000,1.003)*	1.001(1.000,1.003)*	0.999(0.998,1.001)
**NSSI**						
	Hazard Ratio (95% CI) †	ref	0.650(0.352,1.199)	0.291(0.0.131,0.646)	0.513(0.287,0.916)*	1.469(1.050,2.056)*
	Restricted mean survival time at 1120 days (95% CI)					
	Difference—days	ref	0.687(-1.086,2.460)	1.903(0.532,3.274)**	1.617(0.240,2.993)*	-2.879(-4.431, -1.328)***
	Ratio	ref	1.001(0.999,1.002)	1.002(1.000,1.003)**	1.001(1.000,1.003)*	0.997(0.996,0.999)***

† The model included region as a random effect. The variance (standard deviation) of the random effect of region was 0.10 (0.32) for suicide, 0.22(0.47) for suicide attempt, and 0.47(0.69) for NSSI.

*P value <0.05, ** P value <0.01, *** P value <0.001.

Table S27. Non-proportional hazards test for main and subgroup analyses

Suicide		Suicide attempt		NSSI	
**Model**	***P* value †**	**Model**	***P* value †**	**Model**	***P* value †**
hypothesis 1 (Table 2)	0.320	hypothesis 1 (Table 2)	0.790	hypothesis 1 (Table 2)	0.036
hypothesis 2 (Table 3)	0.039	hypothesis 2 (Table 3)	<0.001	hypothesis 2 (Table 3)	<0.001
hypothesis 2 (Figure 2)	<0.001	hypothesis 2 (Figure 2)	<0.001	hypothesis 2 (Figure 2)	<0.001
hypothesis 1 in male (Table S16)	0.110	hypothesis 1 in male (Table S16)	0.580	hypothesis 1 in male (Table S16)	0.260
hypothesis 1 in female (Table S16)	0.260	hypothesis 1 in female (Table S16)	0.910	hypothesis 1 in female (Table S16)	0.062
hypothesis 2 in male (Table S17)	0.490	hypothesis 2 in male (Table S17)	<0.001	hypothesis 2 in male (Table S17)	<0.001
hypothesis 2 in female (Table S18)	0.290	hypothesis 2 in female (Table S18)	0.008	hypothesis 2 in female (Table S18)	<0.001
hypothesis 1 in rural (Table S19)	0.830	hypothesis 1 in rural (Table S19)	0.250	hypothesis 1 in rural (Table S19)	0.024
hypothesis 1 in urban (Table S19)	0.750	hypothesis 1 in urban (Table S19)	0.070	hypothesis 1 in urban (Table S19)	0.940
hypothesis 2 in rural (Table S20)	0.180	hypothesis 2 in rural (Table S20)	<0.001	hypothesis 2 in rural (Table S20)	0.002
hypothesis 2 in urban (Table S21)	0.430	hypothesis 2 in urban (Table S21)	<0.001	hypothesis 2 in urban (Table S21)	0.330
hypothesis 1 aged 15 to 24 (Table S22)	0.330	hypothesis 1 aged 15 to 24 (Table S22)	0.410	hypothesis 1 aged 15 to 24 (Table S22)	0.660
hypothesis 1 aged 25 to 44 (Table S22)	0.200	hypothesis 1 aged 25 to 44 (Table S22)	0.400	hypothesis 1 aged 25 to 44 (Table S22)	0.390
hypothesis 1 aged 45 to 59 (Table S22)	0.160	hypothesis 1 aged 45 to 59 (Table S22)	0.360	hypothesis 1 aged 45 to 59 (Table S22)	0.250
hypothesis 1 aged ≥60 (Table S22)	0.064	hypothesis 1 aged ≥60 (Table S22)	0.021	hypothesis 1 aged ≥60 (Table S22)	0.100
hypothesis 2 aged 15 to 24 (Table S23)	0.440	hypothesis 2 aged 15 to 24 (Table S23)	0.0056	hypothesis 2 aged 15 to 24 (Table S23)	0.340
hypothesis 2 aged 25 to 44 (Table S24)	0.170	hypothesis 2 aged 25 to 44 (Table S24)	<0.001	hypothesis 2 aged 25 to 44 (Table S24)	<0.001
hypothesis 2 aged 45 to 59 (Table S25)	0.620	hypothesis 2 aged 45 to 59 (Table S25)	0.002	hypothesis 2 aged 45 to 59 (Table S25)	<0.001
hypothesis 2 aged ≥60 (Table S26)	0.200	hypothesis 2 aged ≥60 (Table S26)	0.210	hypothesis 2 aged ≥60 (Table S26)	0.200

† The proportional hazards assumption was assessed using Schoenfeld residuals

Table S28. Comparison of characteristics between the individuals with missing values and the included sample

	Missing sample (n = 59,834)	Non-missing sample (n = 185,800)	SMD
**Sociodemographic characteristics**			
Age, mean (SD), y	47.03(14.75)	47.49(14.55)	0.031
Sex			0.125
Male	31,478(52.6)	99,642(53.6)	
Female	28,356(47.4)	86,158(46.4)	
Ethnic			0.105
Han	58,529(97.8)	1,626(0.9)	
Minorities	1,291(2.2)	184,174(99.1)	
Marital status			0.108
Never married	16,369(31.9)	51,157(27.5)	
Married	29,474(57.4)	115,556(62.2)	
Widowed	2,254(4.4)	8,857(4.8)	
Divorced	3,251(6.3)	10,230(5.5)	
Urbanicity			0.063
Rural	35,495(78.5)	150,631(81.1)	
Urban	9,708(21.5)	35,169(18.9)	
Education level			0.099
Primary school or lower	29,314(62.0)	123,532(66.5)	
Middle and high school	16,594(35.1)	58,526(31.5)	
College/university or higher	1,341(2.8)	3,742(2.0)	
Economic situation			0.113
Poverty	22,128(59.1)	120,102(64.6)	

Non-poverty	15,282 (40.9)	65,698 (35.4)	
**Clinical characteristics**			
Family history of psychiatric illness			0.040
Yes	2,037 (3.9)	8,737 (4.7)	
No	50,238 (96.1)	177,063 (95.3)	
Duration of illness			
mean (SD), years	11.35 (10.90)	12.78 (11.17)	0.130
<10	30,001 (50.1)	92,619 (49.8)	0.131
10~19	12,518 (20.9)	48,991 (26.4)	
20~29	6,805 (11.4)	27,489 (14.8)	
⩾30	3,950 (6.6)	16,701 (9.0)	
**History of self-inflicted violence behaviors**			
History of suicide attempt at baseline			0.547
Yes	117 (0.2)	851 (0.5)	
No	43,800 (73.2)	172,068 (92.6)	
Unknown	15,917 (26.6)	12,881 (6.9)	
History of NSSI at baseline			0.536
Yes	150 (0.3)	809 (0.4)	
No	43,715 (73.1)	171,471 (92.3)	
Unknown	15,969 (26.7)	13,520 (7.3)	
**Self-inflicted violence behaviors during follow-up**			
Suicide			0.036
Yes	326 (0.5)	573 (0.3)	
No	59508 (99.5)	185227 (99.7)	

Suicide attempt			0.968
Yes	211 (0.4)	1112 (0.6)	
No	40534 (67.7)	185227 (99.7)	
Unknown	19089 (31.9)	-	
NSSI			0.968
Yes	222 (0.4)	1392 (0.7)	
No	40521 (67.7)	184408 (99.3)	
Unknown	19091 (31.9)	-	

The individuals of missing information on exposure and covariates had a median follow-up of 3.02 years and a maximum follow-up of 12.21 years.
